# Neuroprotective Drug Discovery From Phytochemicals and Metabolites for CNS Viral Infection: A Systems Biology Approach With Clinical and Imaging Validation

**DOI:** 10.3389/fnins.2022.917867

**Published:** 2022-07-25

**Authors:** Anindita Bhattacharjee, Pratik Purohit, Prasun K. Roy

**Affiliations:** School of Bio-Medical Engineering, Indian Institute of Technology (B.H.U.), Varanasi, India

**Keywords:** neurotropic virus infection, phytochemicals, docking study, network pharmacology, MRI fiber tractography, antiviral drug discovery, secondary metabolites, systems biology

## Abstract

**Background:**

Recent studies have reported that pulmo-neurotropic viruses can cause systemic invasion leading to acute respiratory failure and neuroinfection. The tetracycline class of secondary metabolites of microorganisms is effective against several migrating neurotropic viral disorders, as Japanese-Encephalitis (JE), Severe-Acute-Respiratory-Syndrome Coronavirus-2 (SARS-COV2), Human-Immunodeficiency-Virus (HIV), and Simian-Immunodeficiency-Virus (SIV). Another microbial secondary metabolite, cephalosporin, can be used for anti-viral combination therapy. However, a substantial public health debacle is viral resistance to such antibiotics, and, thus, one needs to explore the antiviral efficiency of other secondary metabolites, as phytochemicals. Hence, here, we investigate phytochemicals like podophyllotoxin, chlorogenic acid, naringenin, and quercetin for therapeutic efficiency in neurotropic viral infections.

**Methods:**

To investigate the possibility of the afferent neural pathway of migrating virus in man, MRI scanning was performed on human subjects, whereby the connections between cranial nerves and the brain-stem/limbic-region were assessed by fiber-tractography. Moreover, human clinical-trial assessment (*n* = 140, *p* = 0.028) was done for formulating a quantitative model of antiviral pharmacological intervention. Furthermore, docking studies were performed to identify the binding affinity of phytochemicals toward antiviral targets as (i) host receptor [Angiotensin-converting Enzyme-2], (ii) main protease of SARS-COV2 virus (iii) NS3-Helicase/Nucleoside triphosphatase of Japanese-encephalitis-virus, and the affinities were compared to standard tetracycline and cephalosporin antibiotics. Then, network pharmacology analysis was utilized to identify the possible mechanism of action of those phytochemicals.

**Results:**

Human MRI-tractography analysis showed fiber connectivity, as: (a) Path-1: From the olfactory nerve to the limbic region (2) Path-2: From the peripheral glossopharyngeal nerve and vagus nerves to the midbrain-respiratory-center. Docking studies revealed comparable binding affinity of phytochemicals, tetracycline, and cephalosporin antibiotics toward both (a) virus receptors, (b) host cell receptors where virus-receptor binds. The phytochemicals effectively countered the cytokine storm-induced neuroinflammation, a critical pathogenic pathway. We also found that a systems-biology-based double-hit mathematical bi-exponential model accounts for patient survival-curve under antiviral treatment, thus furnishing a quantitative-clinical framework of secondary metabolite action on virus and host cells.

**Conclusion:**

Due to the current viral resistance to antibiotics, we identified novel phytochemicals that can have clinical therapeutic application to neurotropic virus infection. Based on human MRI scanning and clinical-trial analysis, we demarcated the anatomical pathway and systems-biology-based quantitative formulation of the mechanism of antiviral action.

## Introduction

Central Nervous System (CNS) infection due to neuro-respiratory viruses is prevalent in Southeast Asia, including India. India suffers from diseases caused by neurotropic viruses, such as seasonal Japanese Encephalitis virus (JE), measles virus (MV), herpes virus, human and immunodeficiency virus (HIV), and this scenario was prevalent before the era of severe acute respiratory syndrome coronavirus 2 (SARS-COV2). The World Health Organization (WHO) reported about 500,000,000 confirmed cases of SARS-COV2, including 6,000,000 deaths globally. It is another highly contagious infectious disease after Severe Acute Respiratory Syndrome Coronavirus (SARS-COV) and the Middle East Respiratory Syndrome Coronavirus (MERS-CoV). Respiratory distress is the most common characteristic symptom of COVID-19 (renamed by WHO). However, several neurological signs like anosmia (loss of sense of smell) and ageusia (loss of sense of taste) are also reported in numerous countries as secondary manifestations (Lovato et al., 2020). A cross-sectional study conducted by Printza et al. reported that, out of 182 patients, 38% reported gustatory and 41% olfactory impairment (Printza et al., 2021). Furthermore, COVID-19 contributes to neurological complications, such as seizures, stroke, encephalopathy, and even total paralysis (Fotuhi et al., 2020).

It may be underscored that SARS-COV 1 and 2 have structural similarities, and they have a common binding site angiotensin converting enzyme 2 (ACE2) (Ceccarelli et al., 2020). Current studies have revealed excessive expression of ACE2 receptors in alveolar epithelia cells, mucosa of oral cavity, the intestine, the kidney, and the heart. Recent investigations have also shown that maximum ACE2 receptors are expressed in tongue rather than buccal or gingival oral cavities, indicating vulnerability of oral mucosa toward COVID-19 infection (Xu et al., 2020). Multiple studies on both human and animal models delineate that the central nervous system is a crucial target of SARS-COV (Li et al., 2020), and the virus can enter the brain primarily *via* the olfactory lobe, with rapid spread inside the brain (Netland et al., 2008). The angiotensin-converting enzyme 2 (ACE2) in neurons is the principal binding site for the COVID-19 receptor. It was reported that, after intranasal administration of SARS-COV in transgenic mice that express hACE2, the virus spreads rapidly from airway epithelia to the brain, followed by the infiltration of macrophages and lymphocyte, thereby causing upregulation of cytokines and chemokines in both the lung and the brain (McCray et al., 2007).

In earlier studies, the therapeutic potential of secondary metabolites as minocycline was reported in treating several neurological viral diseases like Human Immunodeficiency Virus (HIV), Japanese Encephalitis (J.E.), Simian Immunodeficiency Virus (SIV) (Dutta and Basu, 2011; Kumar et al., 2015a). The tetracycline class of antibiotics (Minocycline, Doxycycline, Evracycline, Tigecycline) is highly lipophilic (Nikaido and Thanassi, 1993), and so may easily penetrate the lipophilic outer membrane of the SARS-COV2 virus and inhibit the viral RNA replication. These broad-spectrum secondary metabolites exhibit anti-inflammatory and antiapoptotic activities (Giuliani et al., 2005). The minocycline class of antibiotics modifies the functioning of an immune response (Popovic et al., 2002) and exerts neuroprotective actions (Song et al., 2004), which can promote its efficacy in treating COVID19. Moreover, Cephalosporins are also combined with antibiotics to treat viral diseases like influenza (Sutton et al., 2021) and JE (Kumar et al., 2015b). Moreover, in Lyme disease, it is found to be efficacious as doxycycline (Pothineni et al., 2018).

However, antibiotics may possess some drawbacks, such as antibiotic resistance and side effects like fever, nausea, allergic reactions, and diarrhea due to disruption in the normal balance of intestinal flora (Aslam et al., 2018). Globally, the prevalence of resistance with the tetracycline class of drugs is 8.7 and 24.3% for methicillin-resistant Staphylococcus aureus (MRSA) and Streptococcus pneumonia, respectively (Mendes et al., 2015). Therefore, a surge of interest of researchers in plant-based phytochemicals as a possible alternative to antibiotics is taking place to address these issues. Moreover, several reports suggested the noteworthy beneficial role of phytochemicals in virus-mediated neuroinflammation.

Plant-derived phytochemicals are diversified bioactive compounds having various classes, such as terpenoids, flavonoids, alkaloids, and phenols, some of which are used variously as investigational therapeutic agents in neurological disorders. Podophyllotoxin is an aryltetralin-type lignan isolated from perennial herb *Podophyllumhexandrum* that shows comparable potency with the tetracycline class of antibiotics (Malik et al., 2018). Furthermore, chlorogenic acid obtained from plants, such as *Andrographispaniculata, Bixaorellana, Gardenia resinifera, Pongamiapinnata, Sphaeranthusindicus, Solanumtrilobatum, Soyamidafebrifuga*, and *Thespesiapopulnea*, belongs to the hydroxycinnamic acid family and contains caffeic acid and quinic acid, which are reported to have comparable antibacterial, antiviral activity (Luo et al., 2011) and significant β-lactamase inhibitory activity as cephalosporin (Moon, 2015). Naringenin (Alberca et al., 2020) and quercetin (Derosa et al., 2021) are the other potent phytochemicals, predominantly present in *Pongamiapinnata, Thespesiapopulnea, Andrographispaniculata, Psoraleacorylifolia, Soyamidafebrifuga*; these phytochemicals also possess an essential anti-inflammatory role.

However, phytochemicals possessing therapeutic potentials are complex by nature; their potency manifests by targeting multiple targets *via* different phytoconstituents. Therefore, we have taken the network pharmacology approach to investigate the explicit mechanism of phytochemicals for their efficacy in CNS infection. This approach also includes the polypharmacology framework, which helps us replace the customary “one-component/one-target” model with a “multicomponent/multitarget” model. Here, we have found the common targets for both the phytochemicals and viral infections (JE and COVID-19) to identify how aiming at multiple targets can cause an efficacious remediation in the disease process.

Moreover, most of the antimicrobial therapeutic agents used to treat CNS infection require prolonged treatment due to their poor penetrability to CNS, which causes several adverse effects and side effects. Phytochemicals obtained from medicinal plants possess several neuroprotective properties due to their efficient blood brain barrier (BBB) permeability. In this study, a phytochemical of interest, chlorogenic acid, is reported to have significant brain uptake after intravenous administration through the nasal route (Kumar et al., 2019). Naringenin and its glucuronides (metabolites of naringin) exhibited the ability to permeate the BBB into the brain as it has log P value of 2.3 within the acceptable threshold of 1.5–2.7 for a BBB permeable substance (Lawal et al., 2018). Ishisaka et al. reported satisfactory BBB permeability of quercetin through an *in vitro* study with the rat brain capillary endothelial cell line (Ishisaka et al., 2011). Additionally, the highly lipophillic tetracycline class of antibiotics also easily penetrates BBB (Aronson, 1980; Burgos-Ramos et al., 2008).

To underscore, our study is the first human report (as far as we know) to show the following two connections: (i) linkage between gustatory nerves and the respiratory center (ii) linkage between the olfactory nerve and the limbic system areas, and these two connections are demarcated *via* deterministic tracking with fiber tractography so as to provide neuroanatomical validation. These two connected routes can provide the anatomical basis by which viral migration can occur in human retrogradely to the brain *via*: (a) the nasal and buccal routes (for virus lodged in the mouth and the nose), and (b) the vagal route (for virus reaching the lungs). Afterward, our docking studies demonstrated that phytochemicals and the tetracycline class of antibiotics, (especially the newer class flurocycline and glycylcycline, and the standard drug minocycline), have a binding affinity toward (1) main protease of COVID-19, and (2) the binding site of angiotensin-converting enzyme 2 (ACE2) receptor where the SARS-COV2 spike receptor binds. Similarly, the binding affinity of these drugs and phytochemicals with NS3 helicase/nucleoside triphosphatase of Japanese encephalitis is also evaluated. We also evaluated the binding affinity of ceftriaxone (cephalosporin) toward these receptors.

**Figure 1:**
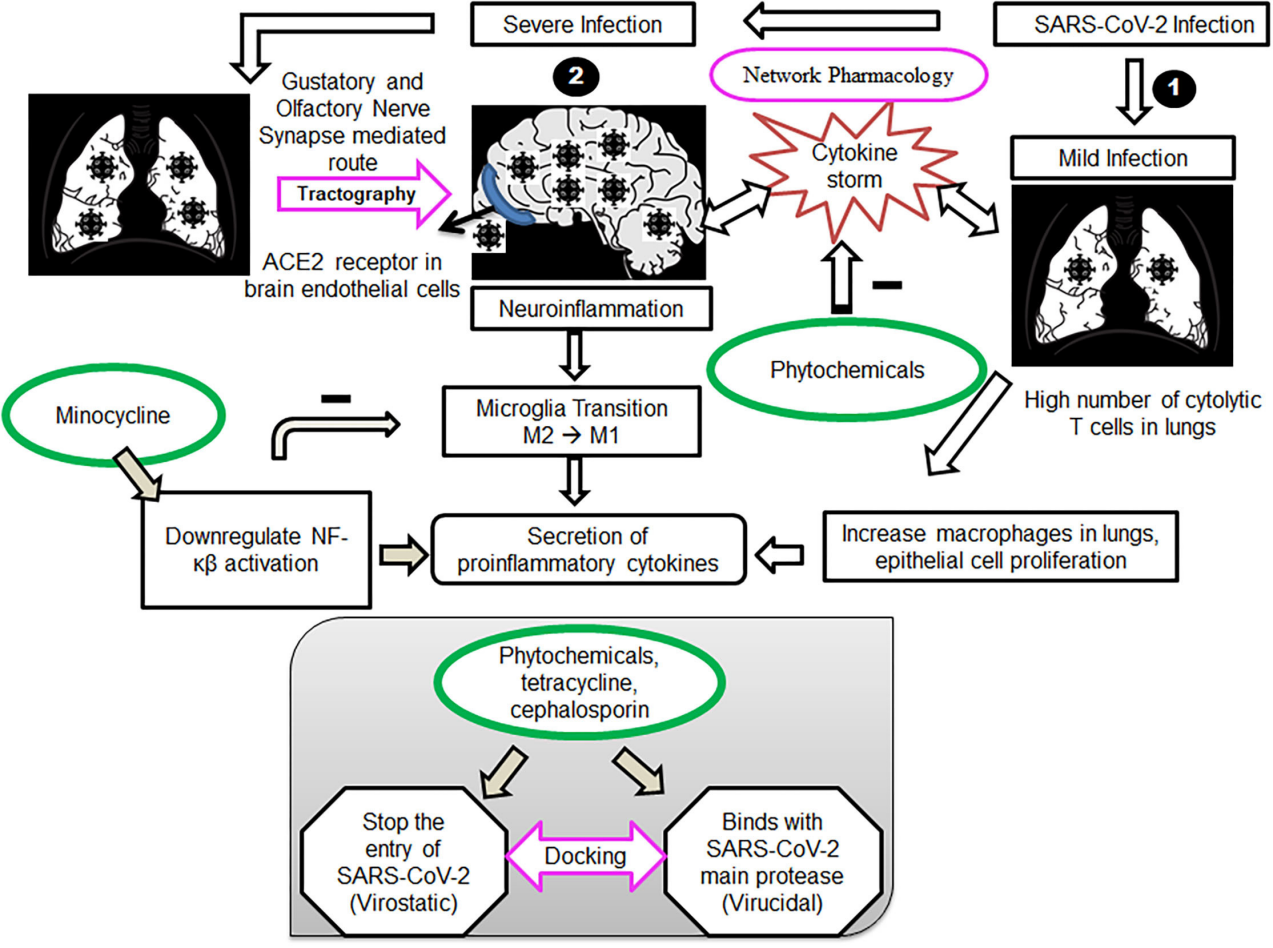
Cell signaling pathways during mild and severe infections with SARS-COV2 and proposed therapeutic intervention of phytochemicals, tetracycline and cephalosporin ligands. (1) Mild infection with SARS-COV2, mainly lungs get affected, causing a high number of cytolytic T cells in the lungs. Phytochemicals act by targeting the T cells and monocytes, driving the cytokine storm in patients, leading to neuroinflammation. (2) Severe infection with SARS-COV2, affecting both lungs and the brain. Virus transmitting through a gustatory and olfactory nerve (tractography)-mediated route, causing neuroinflammation alleviated by Minocycline. Docking studies of phytochemicals, tetracycline and cephalosporin ligands, with the SARS-COV2 spike receptor-binding domain and main protease verify our approach.

Furthermore, we developed a quantitative framework of activity of our proposed drugs on virus and the host cell, analyzed the framework for further enhancement, and developed a predictive platform to identify the newer drugs that would be effective for COVID-19 and similar acute neuro-respiratory viral infections. The procedure is substantiated by data from human clinical trials (Kumar et al., 2015a). Thus, one can consider the drug-receptor interaction to have two components: drug-virus interaction and drug-host cell receptor interaction. Accordingly, our present investigation aims to study the effect of phytochemicals in viral-mediated neuro infections. Therefore, the current study was designed to identify the neuroanatomical pathways through which the virus can cause CNS migration and infection, as well as to examine the specificity and potency of phytoconstituents by performing docking studies. Thereby, we identified the predominant mechanisms through which phytochemicals can show therapeutic efficacy. A quantitative model is also developed to determine the virostatic potency and the virucidal potency (Figure 1).

## Systems Analysis

We have considered two-stage approach in virus physiology, (i) drug-virus receptor interaction and (ii) drug-host cell receptor interaction. Two different drugs can attack the two stages, or a single drug can target both stages.

### Model Formulation

At the initial time *t* = *0*, let there be *N*_0_ receptors, of which *N*_*C*_ is the number of receptors in the host cells; these receptors are the host cell receptors through which the viral material enters (this receptor can also be occupied by a drug, preventing the entry of the virus).

Likewise, *N*_*V*_ is the number of receptors in the viral wall, and if the drug molecule approaches, it can damage the virus.

The total number of receptors *(N)* on which the drugs can act is thus *N* = *N*_*C*_ + *N*_*V*_. If *N* is normalized to 1, then the *N*_*C*_ and *N*_*V*_ can be expressed as a fraction.


(1) $$\begin{array}{r} N_{C}/N+N_{V}/N=N/N=1; \end{array}$$

that is, ^*n*^*C*_0_ + ^*n*^*V*_0_ = *1*

where ^*n*^*C*_0_ and ^*n*^*V*_0_ denote the fractional population of the receptors in the virus and the host cell, respectively, at time *t* = *0*.

Thus, pharmacological action against the virus can be at two sites: (i) the virus coat/virus genome, the drug damaging the virus or, (ii) the host cell receptor, the drug prevents virus entry. In this condition, at the initial time *t*_0_, drug action is on. However, as time elapses, dissipating processes will happen, leading to first-order decay that is the efficacy of the drug-receptor interaction. The well-known process of cytotolerance occurs due to increasing efflux and ejection of the drug from the cell receptors or virus receptors. Hence, as time ensues, the time-varying fractional population of cell receptor ^*n*^*C* (t_1_) would decrease in the first-order or exponential mode as


(2) $$nC(t)={\;}^{n}C_{0}\times \;e^{-\alpha t}n$$

Likewise, the fractional population of the virus receptor


(3) $${\;}^{n}V(t)={\;}^{n}V_{0}\times \;e^{-\beta t}$$

The combined fractional population of both receptors is


(4) $$\begin{array}{r} n\left(t\right)={\;}^{n}C\left(t\right)+{\;}^{n}V\left(t\right)={\;}^{n}C_{0}\times e^{-\alpha t}+{\;}^{n}V_{0}\times e^{-\beta t} \end{array}$$

At the initial time t = 0, the aforesaid equation becomes


(5) $$nC_{0}+{\;}^{n}V_{0}=\;1$$

**Figure 2:**
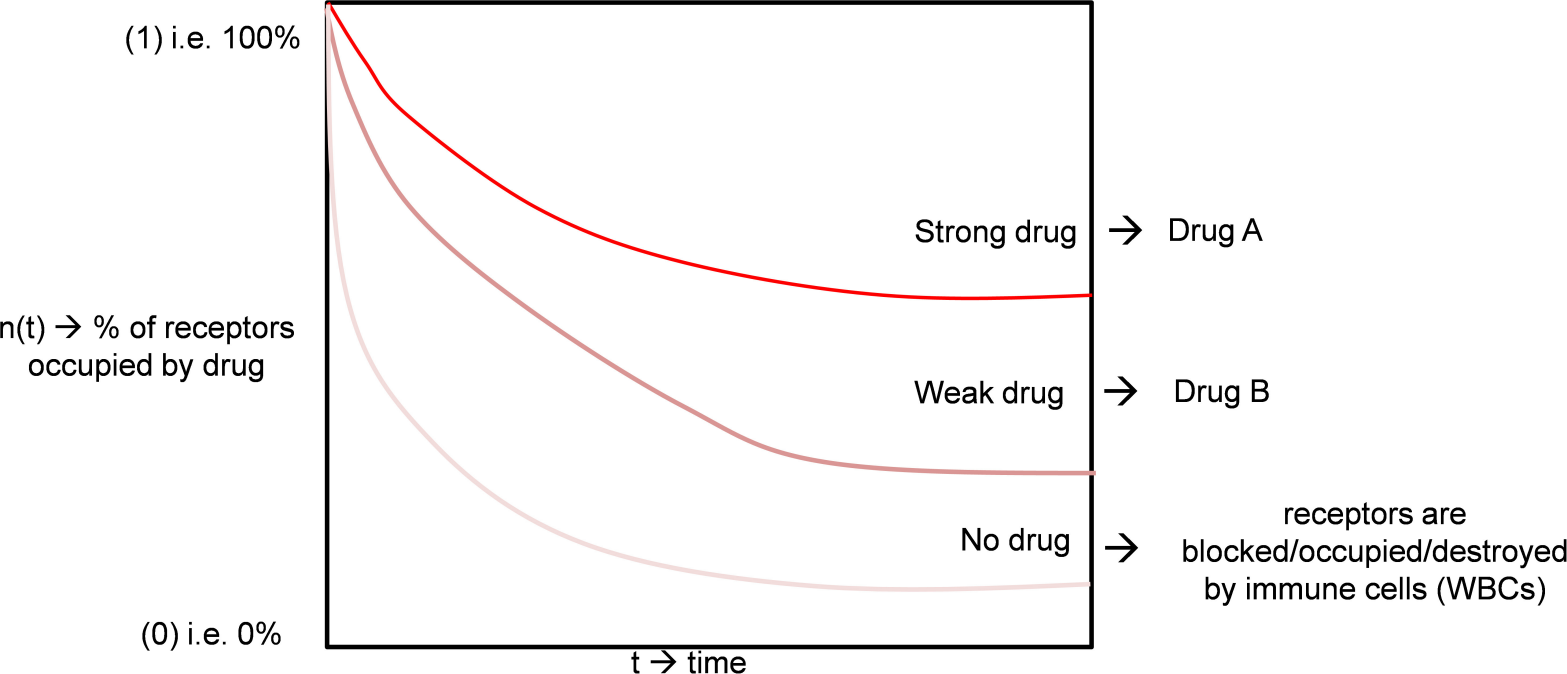
The quantitative model of drug interaction with a viral receptor based on its potency.

Equation (5) may be compared with equation (1). We have plotted an illustrative curve of Equation 4 (Figure 2, Drug A). As the number of viruses is more diminutive than cells, we can take as an instance the fractional virus receptor population, ^*n*^*V* = *0.25*, and fractional cell receptor population ^*n*^*C* = *0.75*. Different drugs can interact differentially on the receptor; one drug may be more effective on the cell receptor. Thus, the viral receptor and relative tolerance of the receptor to the drugs can vary, i.e., all the terms ^*n*^*C*_0_, ^*n*^*V*_0_,α, and β can vary according to the drugs. We show the curves of two drugs (A and B) with different receptor activities (Figure 2).

## Methods

### Image Acquisition

Scanning was done using 3 T magnetic resonance scanners (Philips Achieva 3.0 Tesla at the National Neuroimaging Facility, National Brain Research Center, Manesar, India), a head coil of eight channels, and a SENSE imaging sequence. Inclusion criteria were: Healthy physiological status within normal limits without co-morbidities. For this study, we have included five healthy subjects, and tractography is performed in randomly selected two subjects. Approval for the scanning of the subjects was obtained from the Institutional Human Ethics Committee of National Brain Research Center. The scans were evaluated by the authors having a combined experience of over 25 years in neuroimaging. Detailed acquisition parameters were given in the Supplementary Document (S1).

### Fiber Tracking With DSI Platform

The diffusion MR images are analyses by DSI studio, which is a non-commercial platform. This procedure is used for deterministic fiber tracking, reconstruction, and three-dimensional visualization. (http://dsi-studio.labsolver.org). The restricted diffusion was quantified using restricted diffusion imaging (Yeh et al., 2017). The diffusion data were reconstructed using generalized q-sampling imaging (GQI) with a diffusion sampling length ratio of 1.25 (Yeh et al., 2010). The tensor metrics were calculated. A deterministic fiber tracking algorithm was applied (Yeh et al., 2013). An ROI was placed at pons medulla to show the connections with gustatory nerves. Similarly, another ROI was placed at the hippocampus, entorhinal cortex, and amygdala to show the connections with the olfactory nerve. A seeding region was placed on the whole brain. Additionally, the other parameters like termination index quantitative anisotropy based on Otsu's threshold, angular threshold 60 (Edlow et al., 2012), step size of.10 mm, smoothing of.00, minimum length of 5. mm, maximum length of 300. mm, and seed orientation were kept constant while performing all tracks.

### Docking Studies

#### Retrieval of the Target Protein

The three-dimensional crystal structure of the SARS-COV2 spike receptor-binding domain bound to the ACE2 receptor (PDB ID: 6M0J), the inhibitor bound human angiotensin-converting enzyme-related carboxypeptidase (ACE2) (PDB ID: 1R4L), the N3 inhibitor bound COVID-19 main protease (PDB ID: 6LU7), and JEV NS3 helicase/NTPase (PDB ID: 2Z83) protein were downloaded from the RCSB Protein Data Bank.

#### Selection of Ligands and Its Optimization

The chemical structures of podophyllotoxin, chlorogenic acid, naringenin, quercetin, and tetracycline class (Tetracycline, Minocycline, and Doxycycline) of antibiotics, the newer type of tetracycline like flurocycline (Ervacycline) and glycylcycline (Tigecycline), cephalosporin ceftriaxone, angiotensin-converting enzyme inhibitors MLN-4760, and lisinopril were obtained from the PubChem compound database in the SDF format. Energy minimization of the ligands was done by using the PyRx tool and converted into the PDB format. For the docking study, the ligand was again converted into the PDBQT format by adding charges (Q) and types of bonds (T).

#### Molecular Docking Procedure

The SARS-COV2 spike receptor-binding domain (PDB ID: 6M0J) and the inhibitor bound with the ACE2 receptor (PDB ID: 1R4L) were first removed from the active site of the ACE2 receptor, and the particular amino acid residues involved in this interaction were selected from the string of the ACE2 receptor. Similarly, the N3 inhibitor was also removed from SARS-COV2 main protease to identify the interactive amino acid residues. After that, a suitable grid box was generated, which was large enough to cover all the interacting residues. For the SARS-COV2 spike receptor-binding domain (PDB ID: 6M0J), the center of the grid box was set at 32.977, 31.22, and 19.684 Å, and the box size was set at 68, 88, and 96 Å in *x, y*, and *z* directions, respectively. Similarly, the center of the grid box was set at 39.906, 3.092, and 22.477 Å, and the box size was set at 52, 68, and 60 Å in *x, y*, and *z* directions, respectively, for the inhibitor bound with the ACE2 receptor (PDB ID: 1R4L). For SARS-COV2 main protease (PDB ID: 6LU7), the center of the grid box was set at 15.62, 11.684, and 66.738 Å, and the box size was set at 56, 58, and 64 Å in *x, y*, and *z* directions, respectively. For validation, RMSD value was calculated by superimposing the original co-crystallized ligand for PDB ID: 1R4L, 6LU7, 6M0J, and bound protein for PDB ID: 6M0J and its value were found to be 2.042 Å, 0.362 Å, 2.22 and .066 Å, respectively (Supplementary Figure 1.4, Supplementary Information). AutoDock Version 1.5.6 was used to calculate the ACE2 receptor-ligand interaction using the Lamarckian genetic algorithm (Goodsell et al., 1996). The binding affinity of the ligands toward the COVID-19 binding site of the ACE2 receptor was calculated in AutoDock, which computes the inhibitory constant (Ki) of the receptor-ligand complex. The binding energies generated by AutoDock were, generally, in three forms, namely, intermolecular energy, the total internal energy of the ligand, and torsional free energy. The total of intermolecular and torsional-free energies estimates the free energy of binding, which was then converted into an inhibition constant (Ki), as stated by Hess's law. The lowest value of free-binding energy and its Ki value was used to interpret the binding affinity of the ligands to the receptor. The RMSD of docking protocol is calculated by superimposing the macromolecule obtained from the protein data bank with docked protein.

### Network Pharmacology

#### Retrieval of the Target Genes

Targets of standard drugs and phytochemicals specific to neuroinflammation were retrieved from different databases, such as GeneCard (Safran et al., 2010), Drugbank, and literature review. Moreover, the SARS-COV2 and Japanese Encephalitis target proteins are retrieved from UniProt (https://www.uniprot.org/), GeneCard, and NCBI (Brown et al., 2015). Furthermore, Venn diagram analysis was done to identify the common targets in COVID-19 and JE disease with individual therapeutic agents using the Venny tool.

#### Network Construction and Topological Analysis

Selected target genes common for an individual therapeutic agent with both the COVID-19 and JE virus target genes were imported in Cytoscape plugin, GeneMANIA (Multiple Association Network Integration Algorithm) to construct their network based on their functional relations (Smoot et al., 2011). The resulting networks of genes were then analyzed based on their co-expression, genetic interaction, physical interaction, pathway, colocalization, and predicted and shared protein domains. Furthermore, the CytoNCA plugin was used to study the topological parameters, such as betweenness centrality, degree centrality, and closeness centrality of the resulting network (Tang et al., 2015).

#### Gene Ontology (GO) and KEGG Enrichment Analysis of Targets

Gene ontology is a way to depict detailed information of genes and their products in terms of molecular function (MF), biological process (BP), and cellular component (CC) (Thomas, 2017). We have used the PANTHER (Protein Analysis Through Evolutionary Relationships) tool for GO analysis of selected target genes with their role in different KEGG pathways (Mi et al., 2017). Genes predicted to be involved in the neuroinflammatory response and neurorespiratory viral infection from GO term analysis were compared with CytoNCA-predicted genes. Furthermore, those genes were identified from the Coronavirus disease KEGG pathway (map05171) to understand their functions.

## Results

### MRI Tractography Analysis

#### Connection Between Brain Stem and Gustatory Nerves

**Figure 3:**
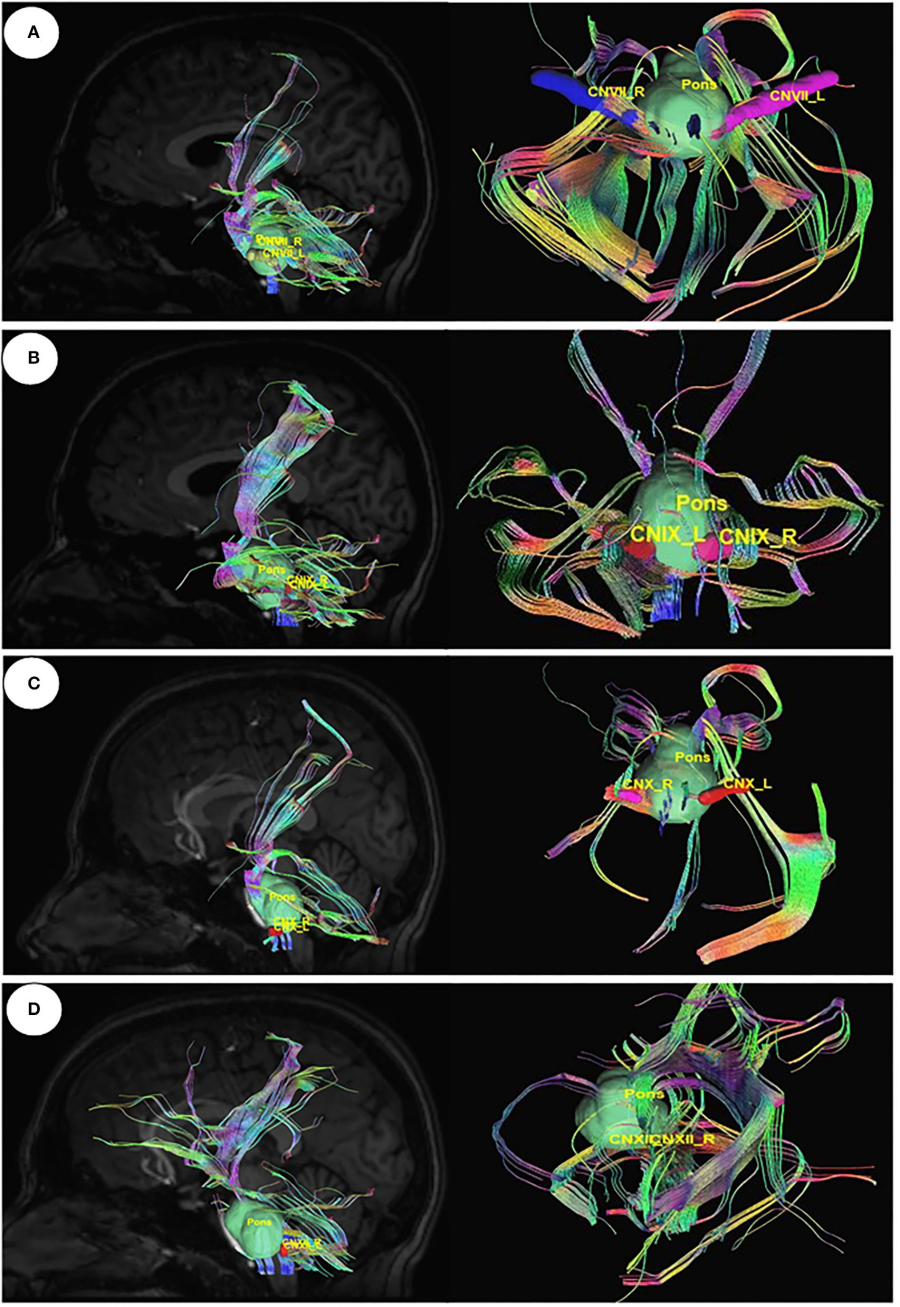
Diffusion MRI fiber tracking-connecting pons with the gustatory nerves. **(A)** pons to cranial nerves VII, **(B)** pons to cranial nerves IX, **(C)** pons to cranial nerves X, and **(D)** pons to cranial nerves XII.

**Figure 4:**
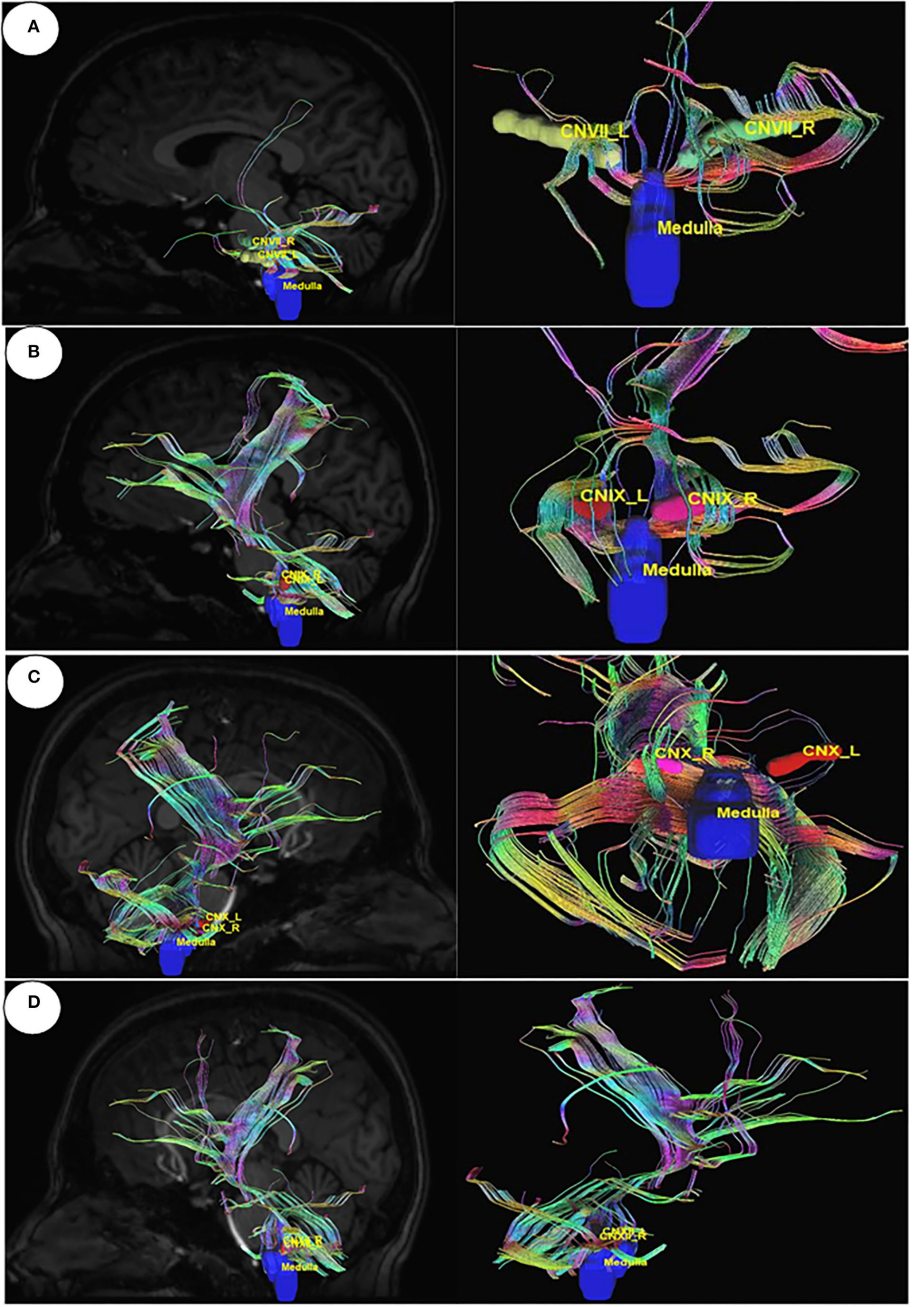
Diffusion MRI fiber tracking connecting medulla oblongata with gustatory nerves. **(A)** medulla to cranial nerve VII, **(B)** medulla to cranial nerve IX, **(C)** medulla to cranial nerve X, and **(D)** medulla to cranial nerve XI.

Utilizing Diffusion MRI fiber tracking, the connections between brain stem (medulla oblongata and pons) where the respiratory center is located and the three gustatory nerves, which are glossopharyngeal (CNIX) that carries taste sensation from the posterior region of the mouth and the throat, facial nerve (CNVII) from the chorda tympani and the vagus nerve (CNX) from the base of the tongue and other parts of the pharyngeal region, were visualized. Besides that, the hypoglossal nerve (CNXII), which innervates the muscles of the tongue, gives tracts with pons. We have obtained the tracts using HCP1021 tractoography atlas to identify the fibers passing through every two ROI pairs (Figures 3, 4).

#### Connection Between Limbic System and Olfactory Nerves

**Figure 5:**
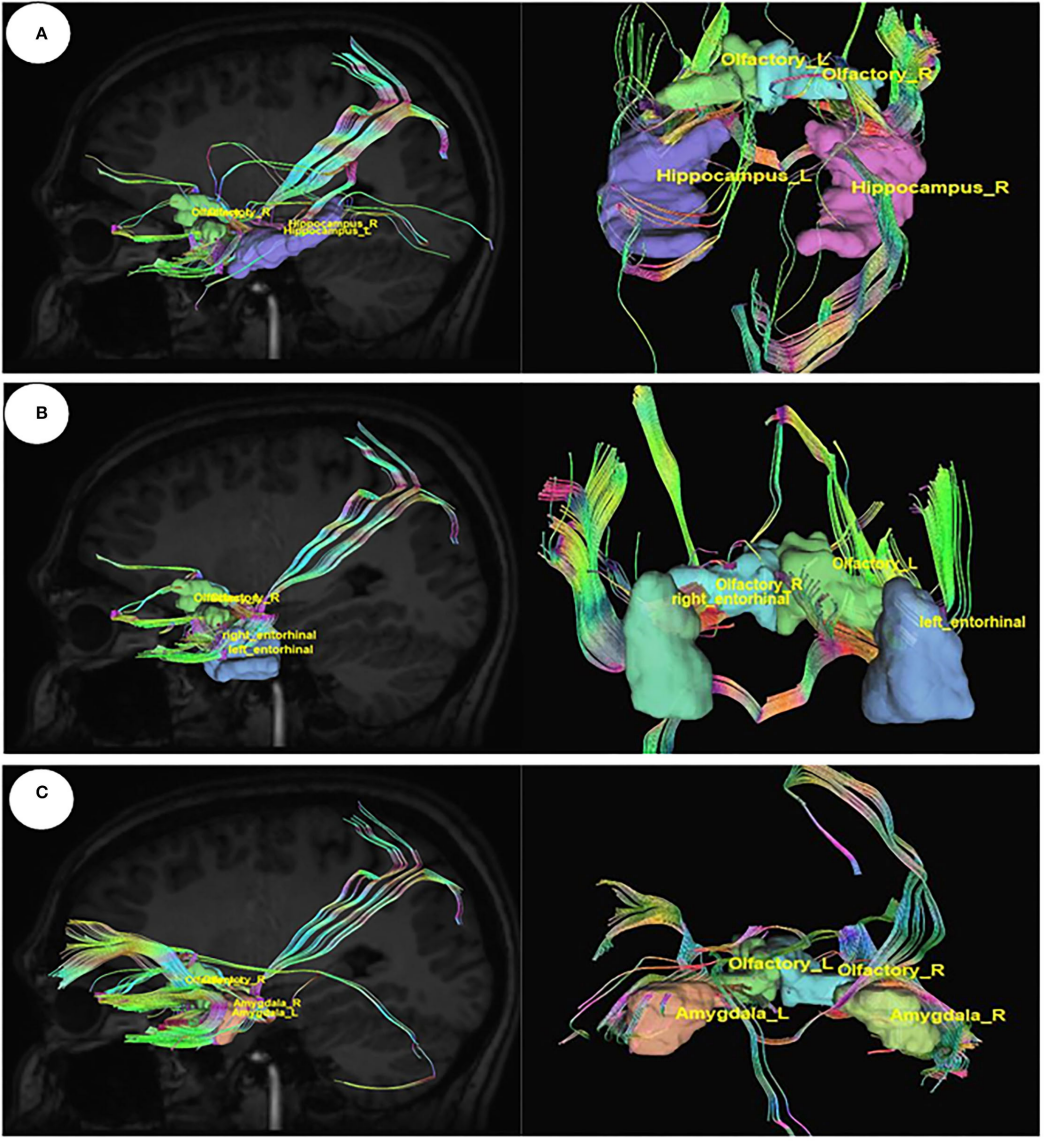
Diffusion MRI fiber tracking connecting the olfactory nerve with **(A)** the hippocampus, **(B)** the entorhinal cortex, and **(C)** the amygdale.

The connections between the olfactory nerve, which is also known as the olfactory tract coming out from the olfactory bulb and the important area of the limbic system hippocampus, the entorhinal cortex, and the amygdala were visualized in DSI studio, and tracts were generated by diffusion MRI fiber tracking (Figure 5).

### Docking Study

#### Binding Sites of ACE Receptor

Severe Acute Respiratory Syndrome Coronavirus 1 and 2 both utilize common receptor angiotensin-converting enzyme 2 (ACE2) for binding. We have studied the binding affinity of the tetracycline class of drugs toward the binding sites of the ACE-2 receptor, where the receptor-binding domain of SARS-COV attached. ACE inhibitors and angiotensin-receptor blockers are used globally to produce beneficial cardiorenal effects (Gavras and Gavras, 1988). Several studies reported that using ACE inhibitors in patients with COVID19 can increase the severity by increasing the expression of ACE2 receptors, which are crucial for virus entry (Vaduganathan et al., 2020).

**Figure 6:**
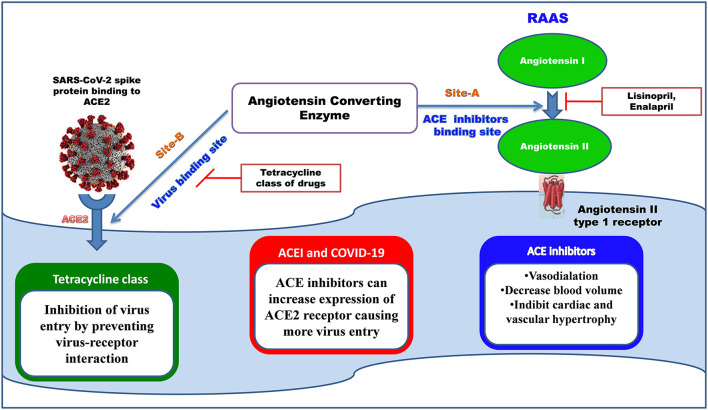
The binding site of ACE inhibitors and SARS-COV2 spike protein.

We have identified that the ACE 2 receptor has two different kinds of binding sites, Site A where the ACE inhibitors like MLN-4760, lisinopril drugs are bound (PDB ID: 1R4L) (Towler et al., 2004), and Site B is the virus-binding site (PDB ID: 6M0J) (Yan et al., 2020) (Figure 6). The two binding sites of angiotensin-converting enzyme (ACE): Site A is the binding site for ACE inhibitors, which helps in preventing the conversion of Angiotensin I to Angiotensin II producing vasodilation, and Site B is the virus-binding site through which viral entry takes place. ACE inhibitors show less-binding affinity toward the virus-binding site (Site-B), where the tetracycline class of drugs shows more negative values of binding energy, indicating the stability of the interaction.

**Figure 7:**
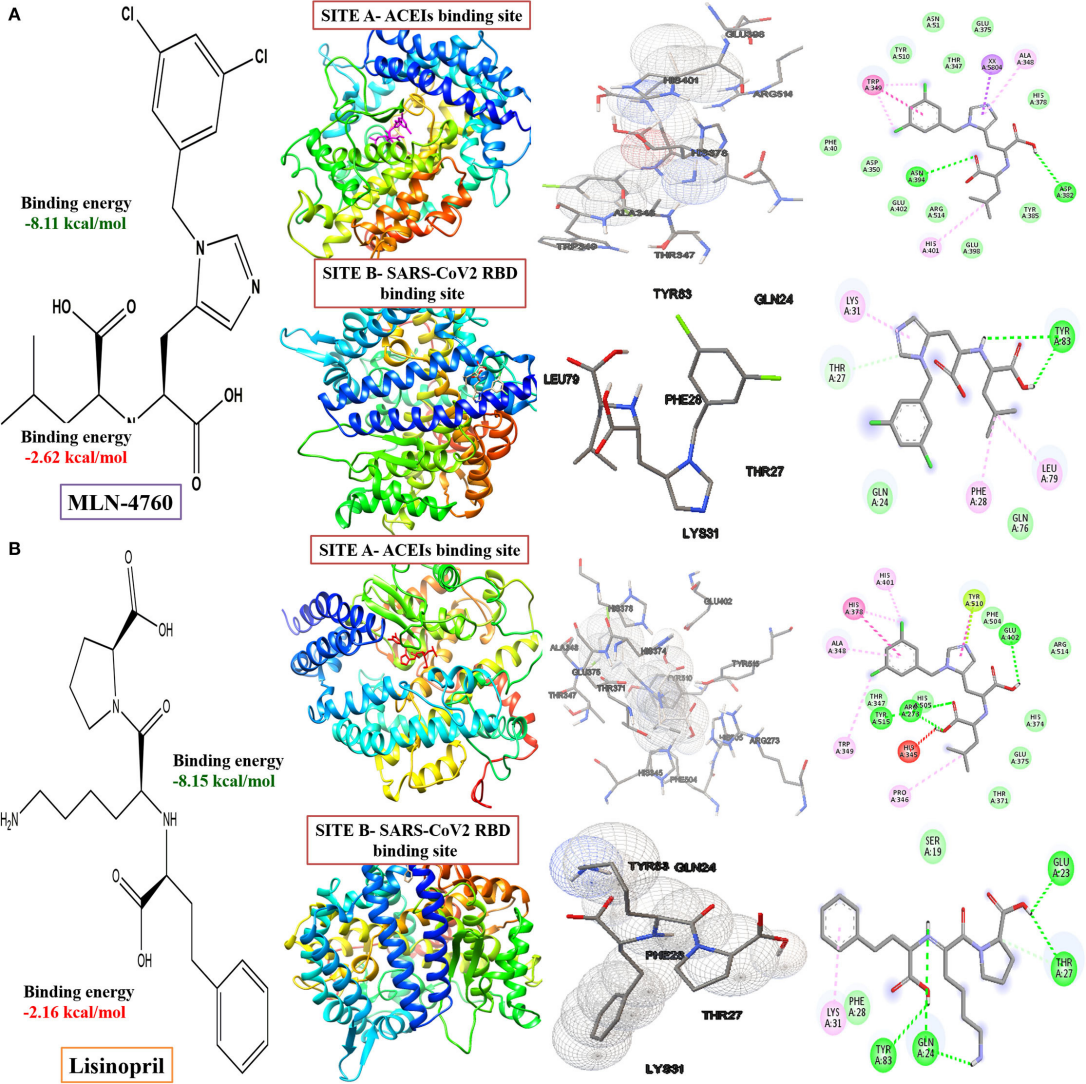
Computed structural comparison, binding features, and the 2D interaction plot (visualization using UCSF Chimera, AutoDock and Biovia discovery studio) of MLN-4760 ((S,S)-2-{1-carboxy-2-[3-(3,5-dichlorobenzyl)-3H-imidazol4-yl]-ethylamino-4 methylpentanoic acid) **(A)** and lisinopril **(B)**, with two binding sites of the ACE-2 receptor (PDB ID: 1R4L and 6M0J, respectively).

Our study revealed that the standard ACE inhibitors had got less binding affinity toward the virus-binding site of the ACE 2 receptor. Docking scores for MLN-4760 and lisinopril are −2.62 kcal/mol and −2.16 kcal/mol in the virus-binding site (Site B), respectively, whereas it is −8.11 kcal/mol and −8.15 kcal/mol, respectively, in the ACE inhibitor-binding site of ACE2 receptor (Figure 7, Supplementary Table S1).

#### Interaction Between ACE2 Receptor and SARS-COV2

**Figure 8:**
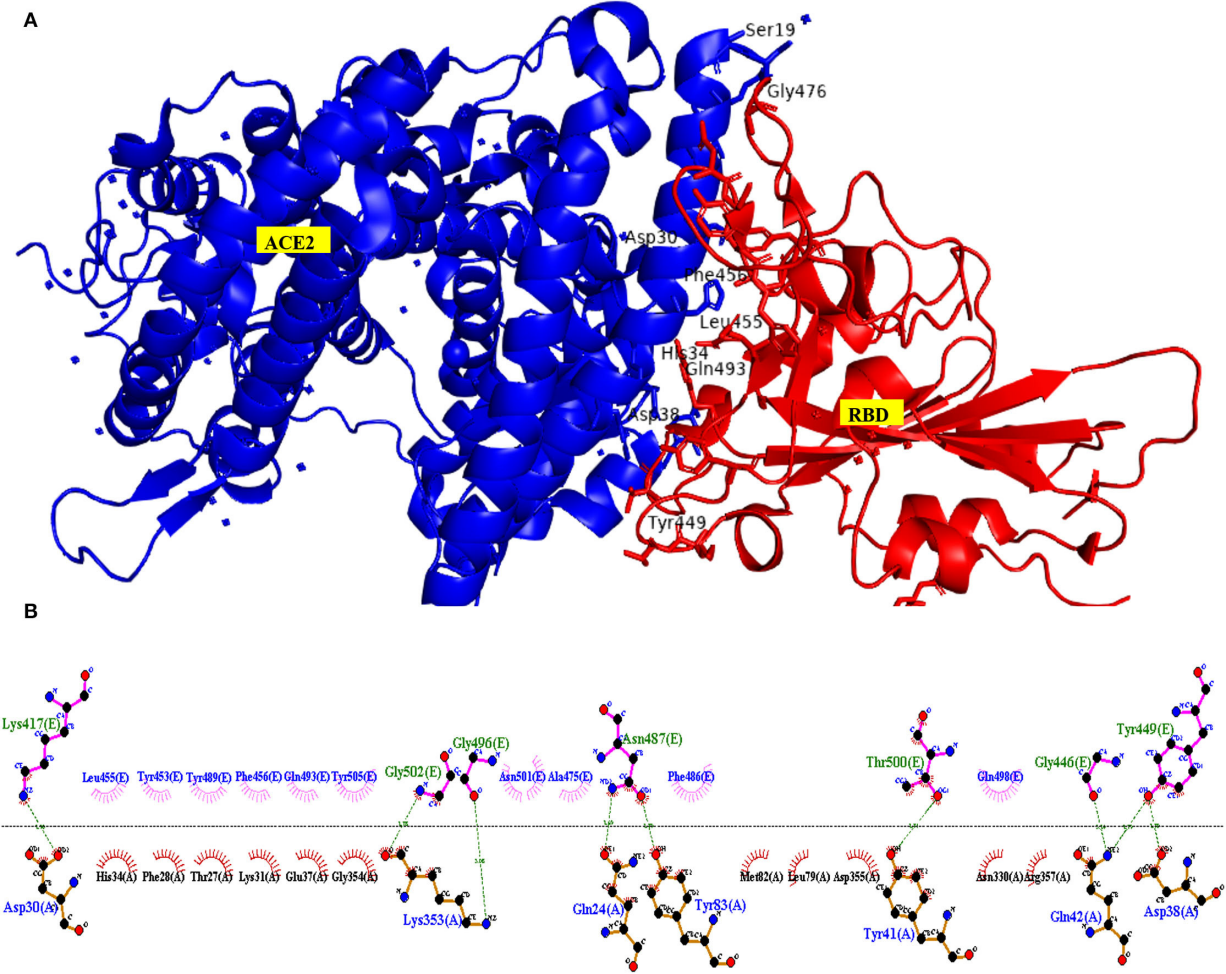
Interacting amino acid residues between the Angiotensin-Converting Enzyme-2 (ACE2) receptor and SARS-COV2 Receptor Binding Domain (RBD) **(A)** and the 2D interaction plot **(B)**, visualized using PyMol and LigPlus.

The critical interacting residues present in the ACE2 receptor, which displayed interaction with the SARS-COV2 spike receptor-binding domain, were divided into three parts: N terminal, a middle portion, and C terminal. A network of hydrogen bonds formed between Gln498, Thr500, and Asn501 of the RBD with Tyr41, Gln42, Lys353, and Arg357 residues of ACE2 receptor, respectively. Lys417 and Tyr453 residues of the middle portion of RBD interact with Asp30 and His34 of the ACE2 receptor, and, at the C terminal, Gln474 of RBD is H-bonded to Gln24 of ACE2, while Phe486 of RBD interacts with Met82 of ACE2 through van der Waals forces, respectively (Lan et al., 2020; Yan et al., 2020) (Figure 8).

#### Interaction Between ACE2 Receptor With Tetracycline Ligands and Phytochemicals

**Figure 9:**
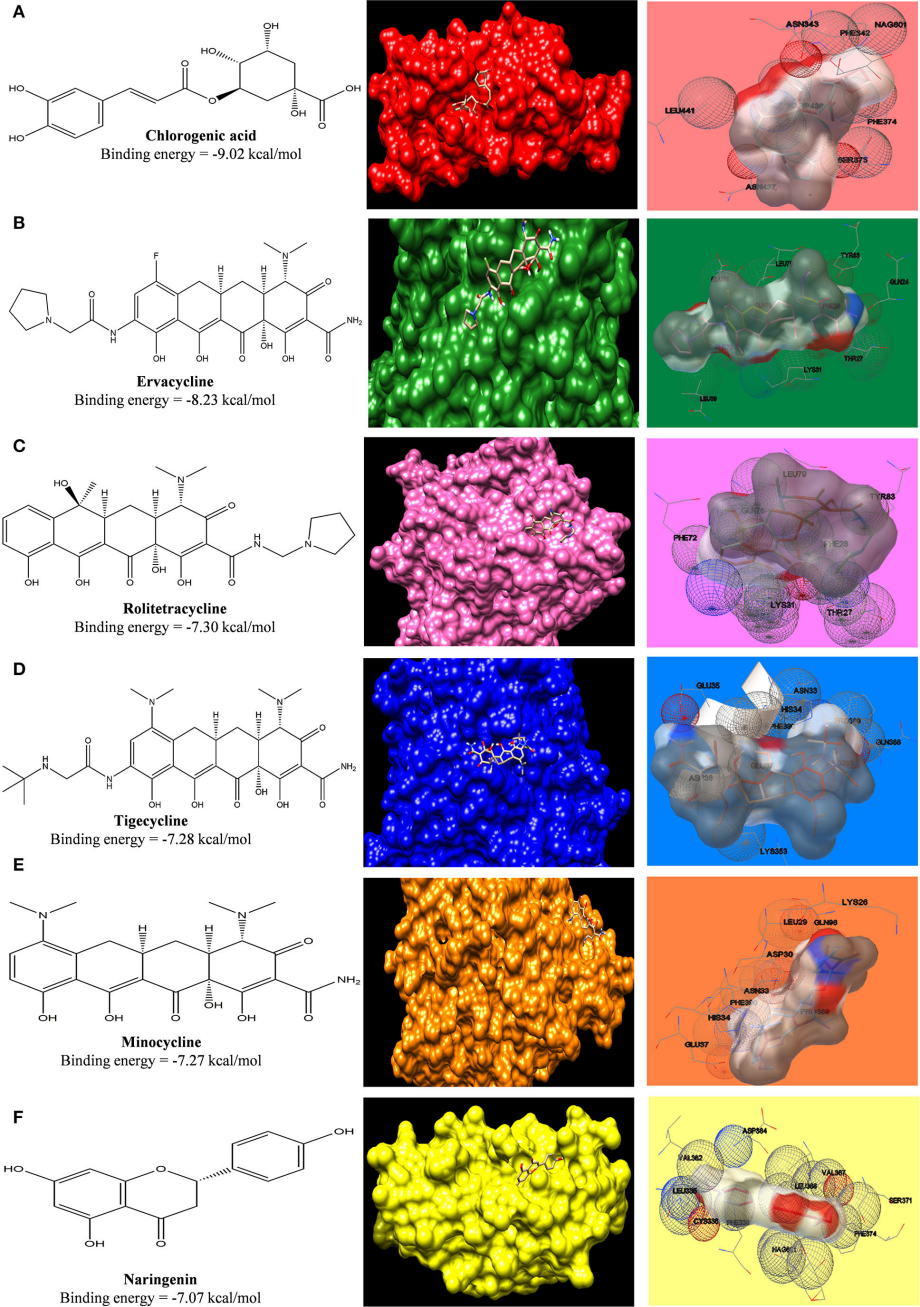
Computed structural comparison and binding features (visualization using UCSF Chimera and AutoDock) of Chlorogenic acid **(A)**, Ervacycline **(B)**, Rolitetracycline **(C)**, Tigecycline **(D)**, Minocycline **(E)**, and Naringenin **(F)** with Angiotensin-Converting Enzyme Receptor 2. The lower the binding energy, the greater will be the binding affinity.

For the docking study, the grid was formed around the interacting residues of the ACE2 receptor, which were Tyr41, Gln42, Lys353, Arg357, Asp30, His34, Gln24, and Met82 (Yan et al., 2020). The interaction of ligands with the receptor is shown in Figure 4, and the free energy of binding along with inhibition constant (Ki) is tabulated in Supplementary Table S2. In our study, we have observed that, in comparison to the standard tetracycline class (Tetracycline, Minocycline, and Doxycycline), phytochemical chlorogenic acid and the newer type of tetracycline-like fluorocycline (Ervacycline) show greater, and glycylcycline (Tigecycline) shows a comparable binding affinity toward the SARS-COV2 virus-binding site of the ACE2 receptor. There were several interactions among the receptor and ligands in which chlorogenic acid interacted with Ser373 and Trp436; minocycline interacted with Asp30 and His34; Naringenin interacted with Phe338 and Cys336; Tetracycline interacted with Gln24 and Met82, Doxycycline with Asp30, Tigecycline with His34, and Ervacycline with Gln24 amino acid residues among other interactions. The amino acid residues mentioned were the residues present in the SARS-COV2 spike receptor-binding-domain-attaching sites of the ACE2 receptor. However, podophyllotoxin showed lower binding affinity with less inhibition constant toward the ACE2 receptor (Figure 9).

#### Interaction Between SARS-COV2 Main Protease With Tetracycline Ligands and Phytochemicals

**Figure 10:**
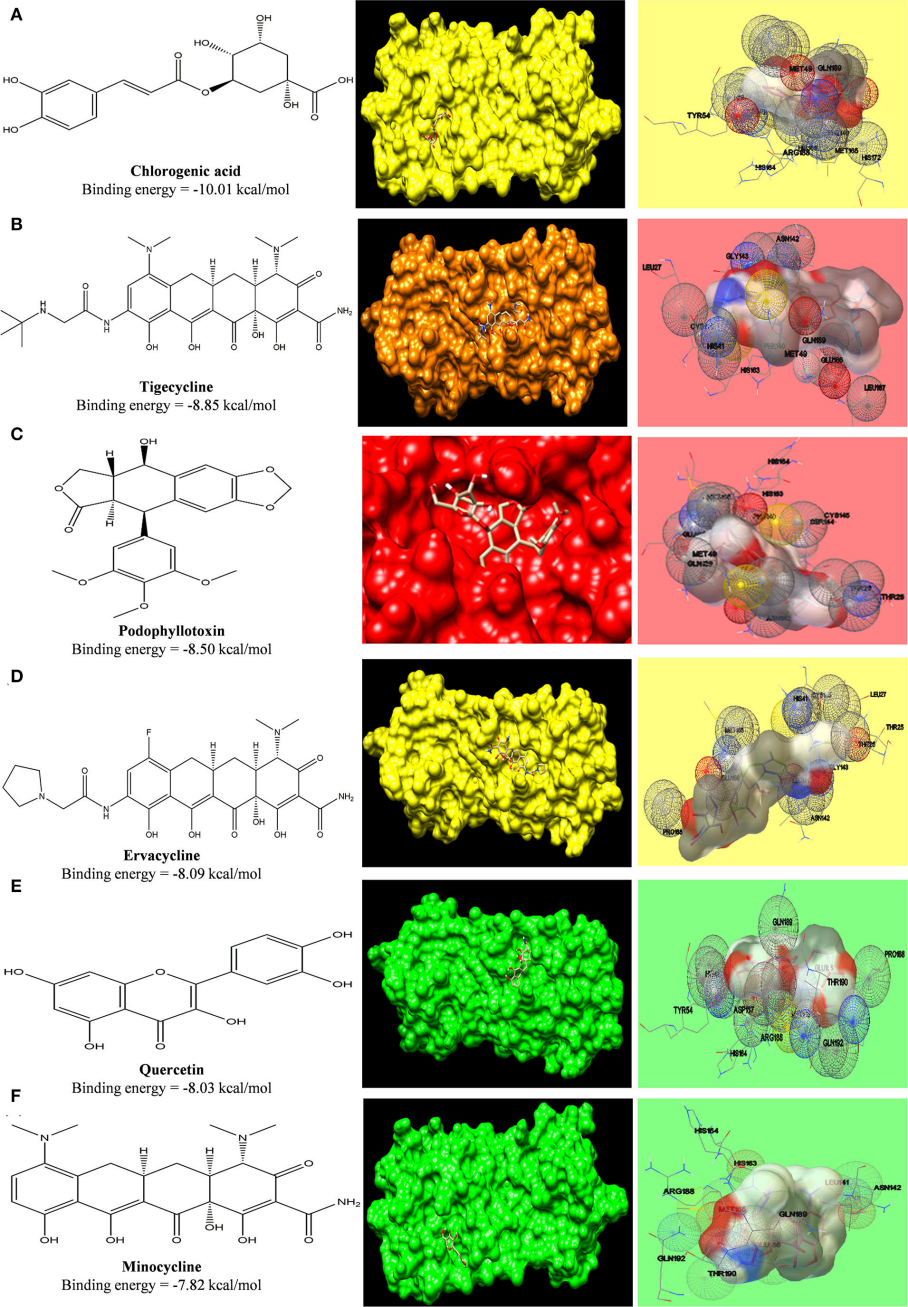
Computed structural comparison and binding features (visualization using UCSF Chimera and AutoDock) of Chlorogenic acid **(A)**, Tigecycline **(B)**, Podophyllotoxin **(C)**, Ervacycline **(D)**, Quercetin **(E)**, and Minocycline **(F)**, with SARS-COV2 main protease. The lower the binding energy, the greater will be the binding affinity.

In a similar fashion, grid was formed around COVID-19 main protease where its potent inhibitor N3 was bound. The interacting amino acid residues of the protease were His41, Cys145, Phe140, Gly143, Pro168, Glu166, Ser144, Met49, and Gln189 (Jin et al., 2020). The interaction of ligands with the receptor is shown in Figure 5, and the free energy of binding along with inhibition constant (Ki) is tabulated in Supplementary Table S3. Phytoconstituent chlorogenic acid, a newer class of antibiotics (Tigecycline and Ervacycline) and podophyllotoxin showed higher binding affinity toward SARS-COV2 main protease compared to standard tetracycline (Minocycline) (Figure 10).

### Validation of Our Data

#### Docking Results With Japanese Encephalitis

**Figure 11:**
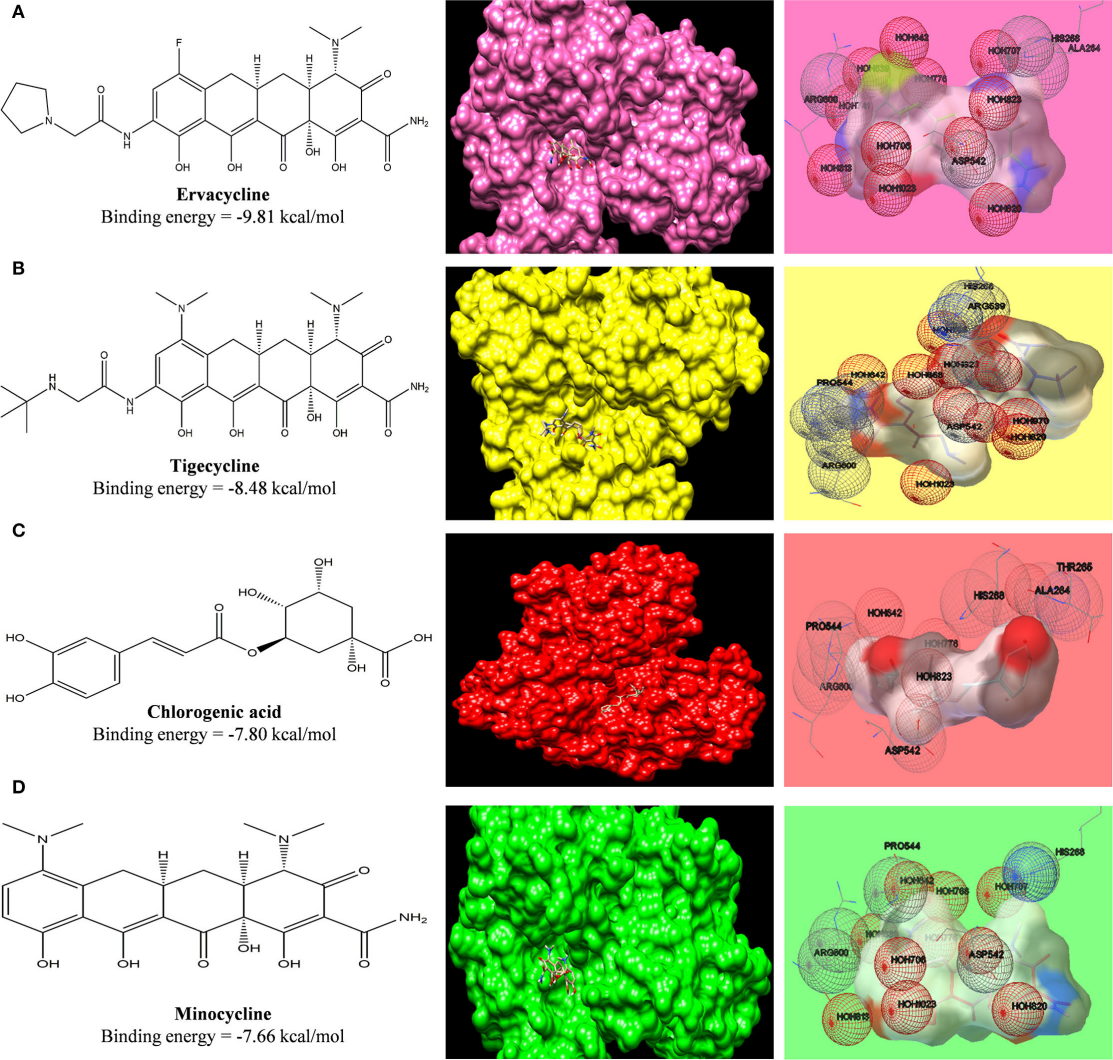
Computed structural comparison and binding features (visualization using UCSF Chimera and AutoDock) of Ervacycline **(A)**, Tigecycline **(B)**, Chlorogenic acid **(C)**, and Minocycline **(D)**, with NS3 helicase/nucleoside triphosphatase of Japanese encephalitis. The lower the binding energy, the greater will be the binding affinity.

A clinical trial of minocycline in treating Japanese encephalitis shows a better outcome (Kumar et al., 2015a). To validate our data on whether the binding affinity of standard tetracyclines, cephalosporins, and phytochemicals toward the SARS-COV2 can be pharmacologically effective or not, we have compared the binding affinity of minocycline with COVID-19 main protease to the binding affinity of minocycline against NS3 helicase/ nucleoside triphosphatase of Japanese encephalitis (J.E.), which possesses enzymatic activities of a serine protease, helicase, and nucleoside 5'-triphosphatase (NTP). In accordance with an earlier docking study done by Nath, M. et al., between minocycline and NS3 helicase/NTPase of J.E. virus, the docking score was around −115.024 kcal/mol calculated *via* iGEMDOCK v2.1 (Nath and Deb, 2018), which was approximately −7.82 kcal/mol when calculated *via* AutoDock 1.5.6. In our study, we have find similar binding affinity of minocycline (−7.66 kcal/mol) and ervacycline, with more negative values of binding-free energy (−9.81 kcal/mol), with SARS-COV2 main protease indicating potential of these agents in treating COVID-19 (Figure 11). Moreover, we have compared the binding affinity of chlorogenic acid toward NS helicase of JE virus (−7.80 kcal/mol), which also revealed equivalent values (Supplementary Table S4). Furthermore, the binding affinity of ceftriaxone (−6.76 kcal/mol) toward NS helicase of JE virus was comparable with other phytochemicals, such as podophyllotoxin (−6.47 kcal/mol), naringenin (−6.16 kcal/mol), and quercetin (−6.13 kcal/mol).

#### Modeling With Clinical Trial Data

We simulate the model (1)–(3), taking data points from the Kaplan Meier Survival curves generated between the Minocycline-treated group and the Ceftriaxone (relative placebo)-treated group in randomized clinical trial of Minocycline in Acute Encephalitis Syndrome carried out by Kumar R et al. (Kumar et al., 2015a).

From equation (4), we can write:


$$\begin{array}{l} n\left(t\right)={\;}^{n}C\left(t\right)+{\;}^{n}V\left(t\right)={\;}^{n}C_{0}\;\times \;e^{-\alpha t}\;+{\;}^{n}V_{0}\times \;e^{-\beta t} \end{array}$$

Analogously, we can write


$$\begin{array}{l} R_{both}^{\%}=R_{\left(cellwall\right)}^{\%}*e^{-\alpha t}+R_{\left(viruswall\right)}^{\%}*e^{-\beta t} \end{array}$$

Here, the following symbols are used:

$R_{\left(\text{cellwall}\right)}^{\%}$ = Percentage of cell wall receptors occupied by drugs

$R_{\left(\text{viruswall}\right)}^{\%}$ = Percentage of viral wall receptors occupied by drugs

*e*^−α*t*^ = Number of cell wall receptors occupied by drug decays with time, as cells learn or develop to efflux or chemotolerance

*e*^−β*t*^ = Number of virus wall receptors occupied by drug decays with time in 1st week (drugs given continuously), as virus learns or develops to efflux or chemotolerance.

**Figure 12:**
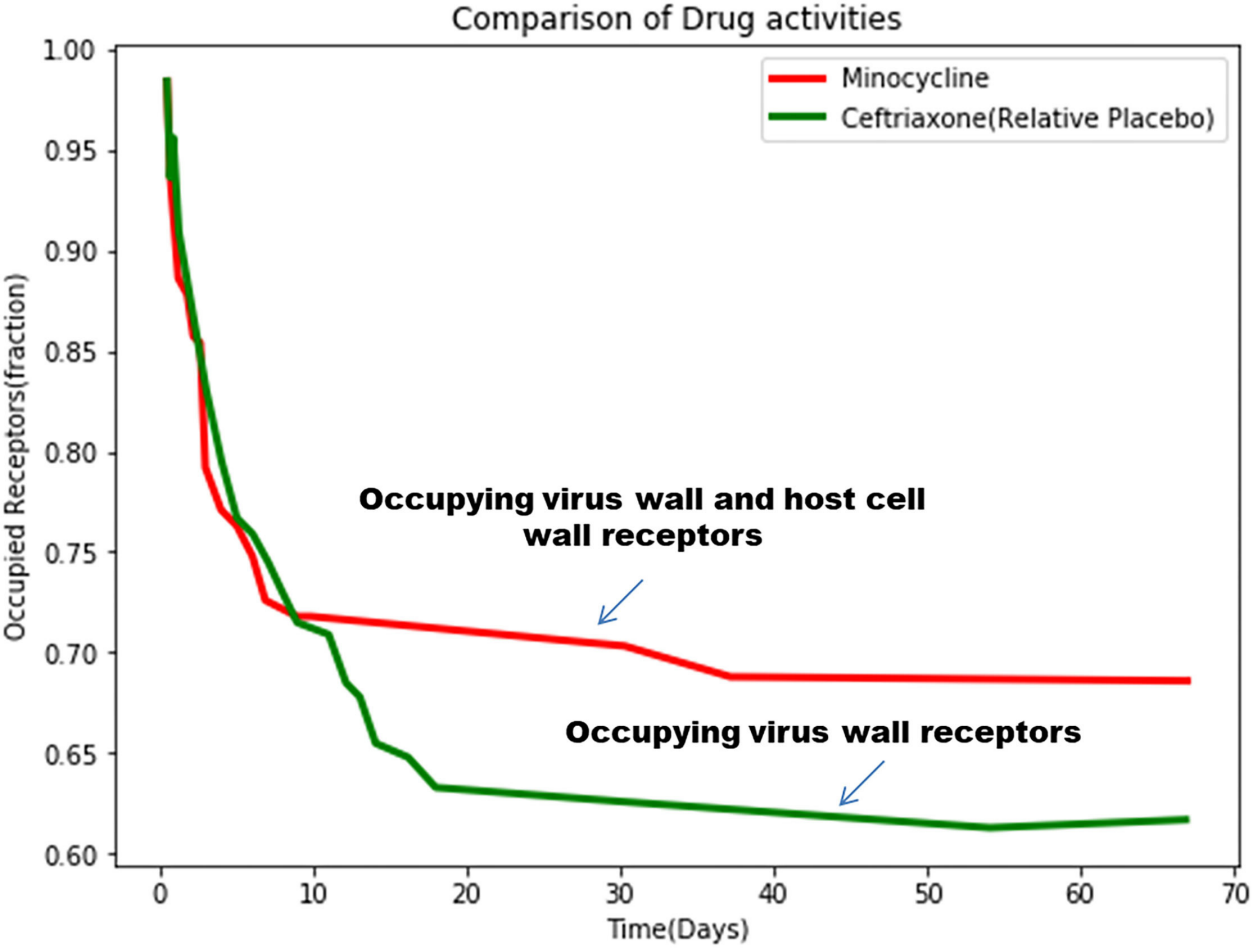
The simulated mathematical model of drug activities toward receptors based on the clinical trial study.

After simulating the equation, we have obtained the values of percentage of receptor occupancy, α and β (Figure 12).

Hence, we obtained:

(i) Interaction of Minocycline With Receptors

(6) $$\begin{array}{r} R_{\text{both}}^{\text{\%}}=0.7044126^{*}exp^{\left(-0.0003975881^{*}t\right)}+0.2955874^{*}exp^{\left(-0.3274611^{*}t\right)} \end{array}$$

*(ii) Interaction of Ceftriaxone (relative placebo) with receptors*

$$\begin{array}{l} R_{\text{both}}^{\text{\%}}=0.6558881^{*}exp^{\left(-0.001120362^{*}t\right)}\;+\;0.3441119{\;}^{*}exp^{\left(-0.2023223^{*}t\right)} \end{array}$$

Comparing these two equations, it can be revealed that ceftriaxone shows predominantly virucidal activity with rapid decaying (α = 0.001120362) compared to minocycline, which shows both virucidal (α = 0.0003975881), as well as virostatic activity (β = 0.3274611), with small values exhibiting slow decay with long-term effects.

### Network Pharmacology Analysis

#### Targets Prediction Results

In this study, two categories of targets (Targets related to SARS-COV2, JE virus, and targets related to phytochemicals, standard drugs) were retrieved from different public databases (Table 1). According to UniProt, GeneCard, and NCBI databases, a total of 248 targets for SARS-COV2 and 346 targets for the JE virus were found, from which the common targets with standard drugs and photochemical were identified by performing a Venn diagram analysis. These common targets were included for further studies.

**Table 1:** Total number of retrieved targets from different databases.

**Category**	**Database**	**Number**	**Total number of unique targets**	**Number of common targets with disease**	**CytoNCA Predicted top 10 genes**
Disease targets					
COVID19	• UniProt • GeneCard • NCBI	• 27 • 239 • 8	248		
JE	• UniProt • GeneCard • NCBI	• 10 • 344 • 9	346		
Drug targets	•	•			
Ceftriaxone	• GeneCard • DrugBank	• 40 • 88	88	24	IFNG, HLA-DRA, CXCL8, IL6, IL10RA, CD4, IL1R1, IL6ST, IL1B, TNF
Chlorogenic acid	• GeneCard • DrugBank	• 25 • 43	68	11	JUN, NFE2L2, MAFK, MTOR, NFE2, MAFG, HIF1A, NOS3, IL1B, ATP2B1
Minocycline	• GeneCard • DrugBank	• 91 • 46	136	39	CCL5, CXCL8, CCL2, STAT1, IFNG, CCL18, CXCL9, CXCL10, CCR5, CCL4
Naringenin	• GeneCard • DrugBank	• 49 • 48	97	18	TP53, STAT3, FAS, AKT1, STAT1, JUN, TNFRSF1A, FADD, CREBBP, FASLG
Podophyllotoxin	• GeneCard • DrugBank	• 44 • 48	90	23	STAT1, IRF2, IRF1, STAT3, IFNG, ITGB2, ICAM1, JUN, IRF9, IRF3
Quercetin	• GeneCard • DrugBank	• 183 • 48	226	79	JUN, CXCL8, STAT1, IFNG, CCL2, STAT3, IRF1, CCL5, IL6, CXCL1

#### Gene–Gene Interaction Analysis

**Figure 13:**
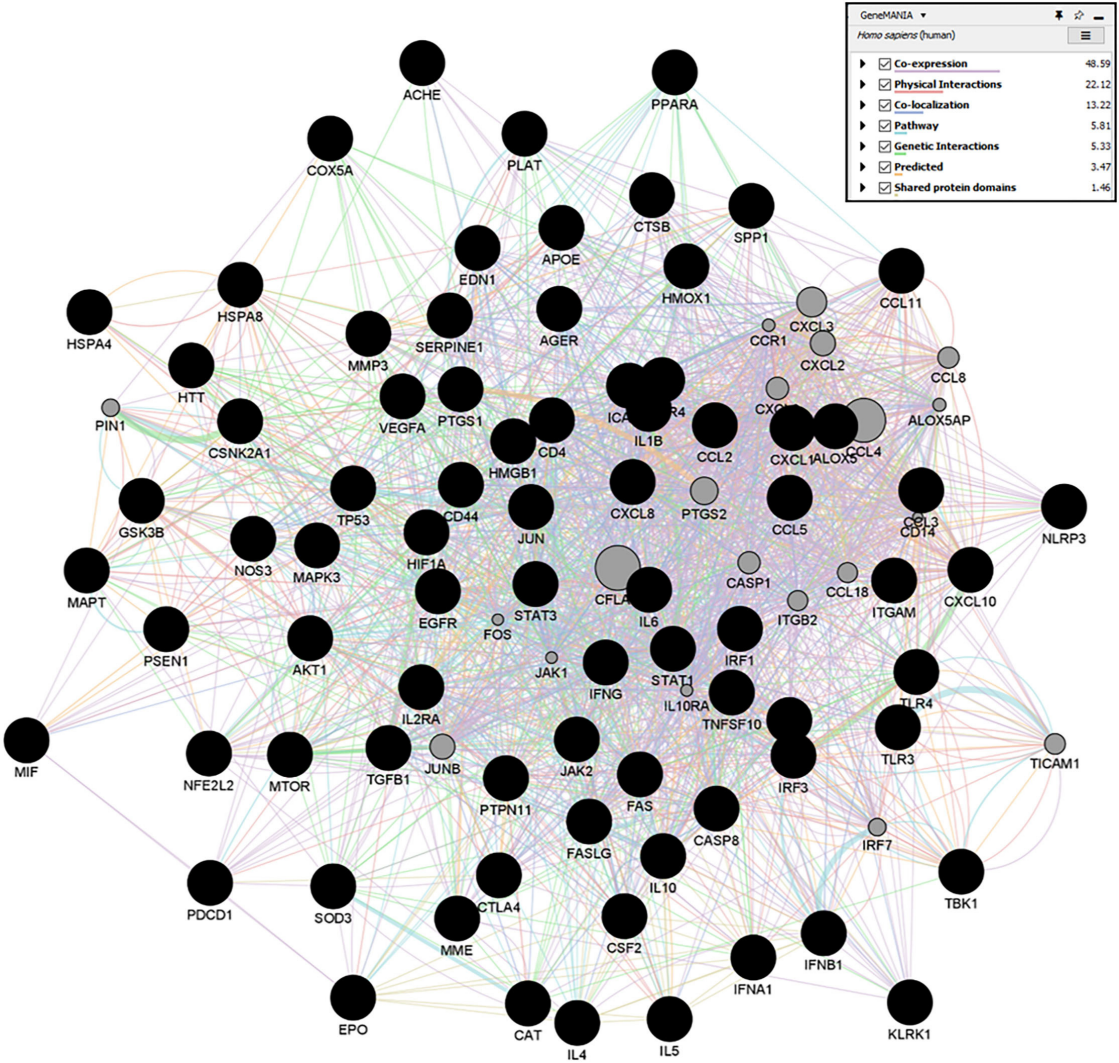
GeneMANIA networks showing the gene-gene interaction results of Quercetin targets. Network displays the strength of interaction (edge thickness), type of interaction (colors), many edges in between nodes, and the score (size of node).

A Cytoscape plugin, GeneMania, was used to construct the gene-gene interaction network of targets of individual therapeutic agents (Figure 13). Circular nodes denoted genes, and colored edges presented their different correlations. Larger circles denoted that these genes were most correlated to other genes in this network. For detailed information of all the gene-gene interactions of individual agents, refer to Supplementary Figures 1A–E). Furthermore, topological parameters, Betweenness Centrality, Degree Centrality, and Closeness Centrality of each node, were observed. Degree Centrality is the measure of direct connections of a node in the network, and a higher degree indicates the high impact of that node. Based on the highest degree score, we selected the top10 nodes of the network.

#### Gene Ontology and KEGG Enrichment Analysis for Targets

We have used PANTHER to obtain several Biological Processes (BP), Molecular Functions (MF), Cellular components (CC), Protein class, and pathways for ten key targets (Table 1) predicted in CytoNCA. In the case of BP, targets were enriched in different biological regulations, cellular processes, immune system processes, response to stimulus, and signaling. For CC, targets were enriched in the cellular, anatomical entity, intracellular, and protein-containing complex. In the case of MF, the targets were involved in binding, molecular function regulator, and molecular transducer activity. The targets were observed to be enriched in different protein classes like a defense/immunity protein, a intercellular signal molecule, and a transmembrane signal receptor.

**Figure 14:**
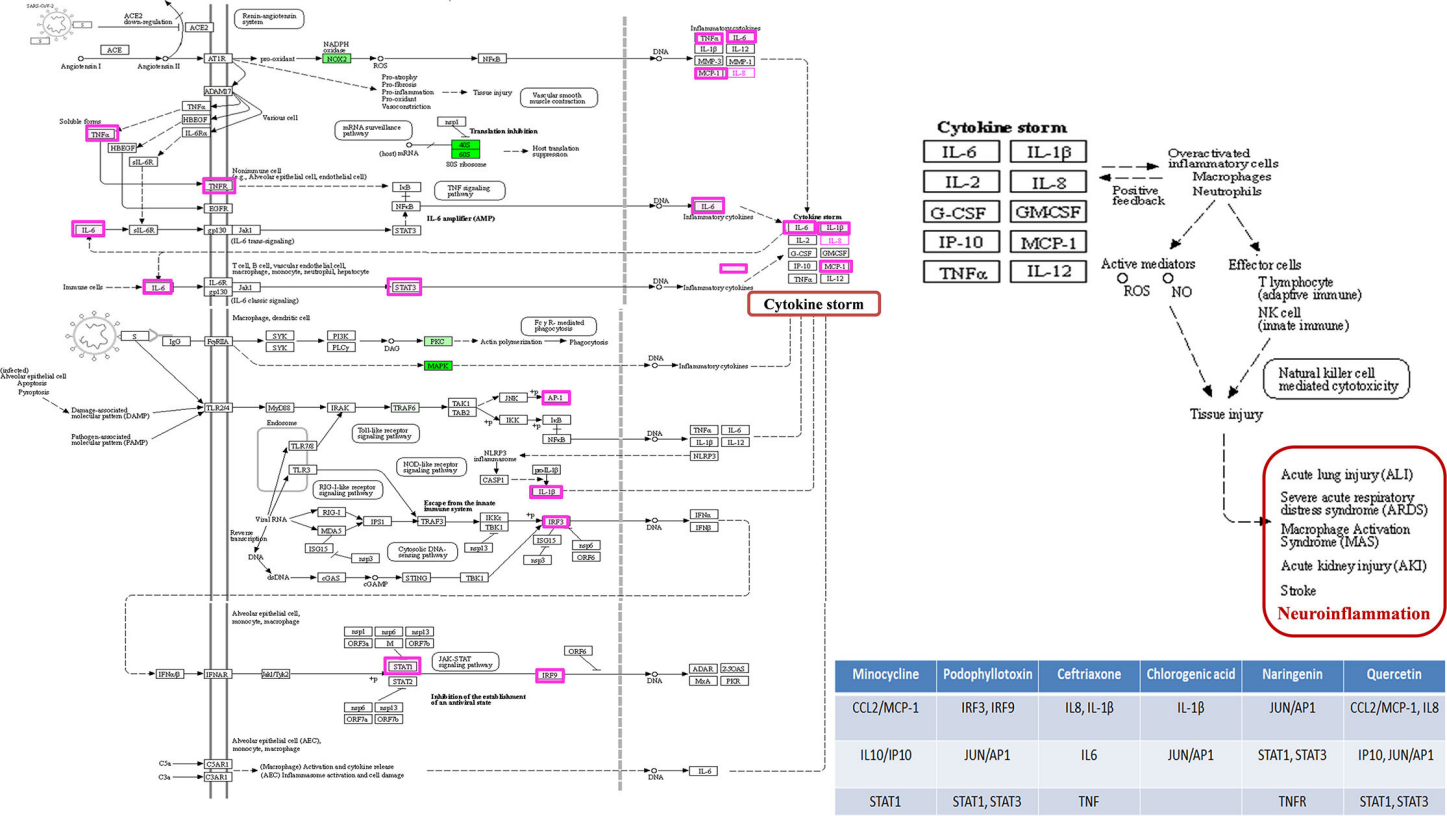
The KEGG pathway of Coronavirus Disease. The critical genes identified with CytoNCA are highlighted, and their role in cytokine storm-mediated neuroinflammation is depicted.

Pathways analysis of targets was enriched in Angiogenesis, B cell activation, Hypoxia response *via* HIF activation, Inflammation mediated by the chemokine- and cytokine-signaling pathway, the Interleukin signaling pathway, the JAK/STAT signaling pathway, T cell activation, and the Toll receptor signaling pathway. For more detailed results of all the categories, refer to Supplementary Material (Supplementary Table S5). Inflammation mediated by chemokine- and cytokine- signaling pathways was the highest intensity in the gene ontology analysis, which substantiates that all the therapeutic agents targeted the cytostorm-mediated neuroinflammation during CNS infection. Moreover, the role of those critical genes in COVID-19 infection was also determined from the KEGG pathway (map05171) of Coronavirus disease (Figure 14).

## Discussion

The current epidemic of positive-sense single-stranded RNA virus produces some of the most lethal public health emergencies worldwide, e.g., MERS, SARS-1, COVID19, and Japanese Encephalitis. These viruses induce pan-systemic infection, and the death of patients occurs due to cytotoxic shock syndrome and central cardiorespiratory failure, i.e., dysfunction of the neural cardiorespiratory center in the medulla oblongata and pons. These viruses are also neurotropic, and we delineate that they infect the respiratory center of the brain (medulla) through anterograde transmission from nasal olfactory or pharyngeal cranial nerves and through retrograde transmission from the lung epithelium *via* the vagus nerve. Our findings were corroborated by other multiple studies where there were hyper-intense MRI signal changes in pons (Paterson et al., 2020) in patients with COVID19, while penetration of SARS-COV2 through olfactory mucosa to medulla oblongata was also reported (Meinhardt et al., 2021).

Some anti-viral pharmacological agents act by two mechanisms (i) virucidal activity: the agent directly damages the virus coat or its RNA [drug-virus receptor interaction], and (ii) virustatic activity: the agent binds on the host cell receptor through which the virus enters, thus preventing virus entry into the host cell (Drug-Cell receptor interaction).

Additionally, we have developed a systems-biology-based double-hit mathematical bi-exponential model, which integrates these two aspects (virucidal and virustatic effects), and we validate this model by clinical findings of patient survival curves under antiviral treatment. This systems biology model furnishes a quantitative-clinical framework of action of phytochemicals and secondary metabolites on virus and host cells. Furthermore, multiple drugs can be given to modify the processes of cytotoxic shock syndrome. We have shown how phytochemicals and secondary metabolites can be explored for a meaningful therapeutic approach to such CNS infections.

In the present study, we have identified the nerve fiber tracts that enable the connection between gustatory nerves and the olfactory nerve with the respiratory center and limbic system, respectively—–these connections enable the centripetal spread of the virus from peripheral neural epithelium to the brain, thus predisposing to central cardiorespiratory failure in the brainstem region. Afterward, we have shown the binding affinity of the tetracycline drugs, the cephalosporins (ceftriaxone) and phytochemicals, with the Angiotensin-Converting Enzyme Receptor 2, which is the binding site for the SARS-COV2 spike (S) receptor-binding domain, as well as for COVID19 virus main protease that takes part in viral replication and transcription.

In our study, we evaluated the efficacy of phytochemicals along with secondary metabolites (as antibiotics) against viral infection-mediated CNS inflammation. From the Docking studies, we have identified that the phytochemical, chlorogenic acid, as well as the newer class of tetracycline, ervacycline (a fluorocycline), showed more binding affinity compared to the standard tetracyclines, as minocycline, or doxycycline, toward the SARS-COV2 receptor-binding site of the ACE2 receptor.

Furthermore, a phytochemical spectrum, as chlorogenic acid, podophyllotoxin, and quercetin, interacts with Gln189, Met49, Phe140, Glu166, Asn142 residues, which revealed the robust binding affinity of these phytochemicals toward SARS-COV2 main protease, and is comparable with a newer class of tetracyclines, namely, tigecycline and ervacycline. Furthermore, naringenin and quercetin bind with Cys336 residue, and podophyllotoxin binds with Cys480 residue of the receptor-binding domain; these cysteine residues stabilize the domain. Although binding affinity reveals that chlorogenic acid, ervacycline, and tigecycline were promising targets, the standard drug minocycline and tetracycline show more common interactions with SARS-COV2 spike protein against the ACE2 receptor. Moreover, we delineate that minocycline binds with the Asp30 and His34 amino acid residues in the SARS-COV2 spike protein, while tetracycline binds with the Gln24 and Met82 residues in that spike protein. However, ceftriaxone showed less binding affinity toward the host receptor and more toward COVID-19 protease, demarcating its predominant virucidal and less virustatic activity.

Considerable evidence indicates the therapeutic efficacy of minocycline in treating several viral infections like Japanese Encephalitis, West Nile virus, Human Immunodeficiency Virus, Rabies, and Reovirus (Zink et al., 2005; Michaelis et al., 2007; Mishra and Basu, 2008). The anti-inflammatory and neuroprotective role of this broad-spectrum antibiotic was found to be effective in combating these neuroinvasive viruses. Independent of its antibiotic mechanism, minocycline can inhibit toxic microglia (M1) and activate neuroprotective microglia (M2), which leads to suppression of NF-κβ activity, consequently preventing the production of pro-inflammatory cytokines——these are responsible for extreme acute respiratory distress symptom in patients with COVID19 (Zhao et al., 2015; Alam et al., 2016). Our network pharmacology analysis revealed that minocycline could target the pro-inflammatory chemokines (CCL2) and cytokines, whose abrupt increase is referred to as cytokine storms (Narayanappa et al., 2021). Furthermore, it was reported that minocycline regulates endogenous matrix metalloprotease (the MMP-2 and 9 pathway) (Zakeri and Wright, 2008), whereas murine coronavirus utilizes host zinc metalloproteases for entry, survival, and cell-cell fusion (Humar et al., 2004; Phillips et al., 2017).

In addition to synthetic antibiotics, we observed that some phytochemicals substantially possess equivalent potency of antibiotics reported in alleviating CNS infection. To compare, Ding Y et al. reported the antiviral potential of chlorogenic acid against the influenza virus (Ding et al., 2017), and the acid has also β-lactamase inhibitory potency, which is comparable with standard cephalosporins (Moon, 2015). Several reports suggested that chlorogenic acid has shown therapeutic efficacy in SARS-COV2 infection by modulating inflammatory responses (Wang et al., 2021). To corroborate, our gene ontology analysis identified that chlorogenic acid could target neuroinflammation mediated by chemokine and cytokine signaling pathways. It has also been revealed that phytochemicals, as naringenin (Alberca et al., 2020) and quercetin (Derosa et al., 2021), also show antiviral and anti-inflammatory properties, like reducing viral replication and interacting with downstream signaling molecules of Toll-like receptors (TLRs), as well as the JAK-STAT pathway. Furthermore, we inferred that podophyllotoxin has equivalent therapeutic potential as teracyclines (Malik et al., 2018), besides proving to be effective against cytokine storm in patients with COVID-19 (Shah et al., 2021). Our findings suggest that podophyllotoxin could also target IFN-regulatory Factors 3 and 9, which are crucial in SARS-COV-mediated evasion of host innate immune response (Shah et al., 2020).

## Conclusion

The crucial findings of our investigations suggest that phytochemicals and secondary metabolites have both virostatic and virucidal mechanisms to ameliorate CNS infection. To exemplify, phytochemicals as podophyllotoxin and quercetin manifested a greater binding affinity toward the virus protease, suggesting their strong virucidal mode of action. Moreover, our network pharmacology approach substantiates the possible mechanism through which the phytochemicals and secondary metabolites have seminal possibilities in alleviating CNS infection. A noteworthy finding is that ascending viral infection, migrating retrogradely *via* nerve fibers, can induce respiratory failure by affecting the midbrain, and hence phytochemicals, which have a satisfactory CNS permeation profile, may offer a novel therapeutic avenue to resistant neurotropic viral infections.

## Data Availability Statement

The original contributions presented in the study are included in the article/Supplementary Material, further inquiries can be directed to the corresponding author.

## Ethics Statement

The studies involving human participants were reviewed and approved by Institutional Human Ethics Committee of National Brain Research Center, Manesar, Haryana, India. The patients/participants provided their written informed consent to participate in this study.

## Author Contributions

Conceived and designed the experiment, performed the mathematical analysis, bioinformatics study, imaging investigation, wrote the manuscript, and contribution to the article and approval of the submitted version: AB, PP, and PR. All authors contributed to the article and approved the submitted version.

## Conflict of Interest

The authors declare that the research was conducted in the absence of any commercial or financial relationships that could be construed as a potential conflict of interest.

## Publisher's Note

All claims expressed in this article are solely those of the authors and do not necessarily represent those of their affiliated organizations, or those of the publisher, the editors and the reviewers. Any product that may be evaluated in this article, or claim that may be made by its manufacturer, is not guaranteed or endorsed by the publisher.
