# Supplementary material for: Neuroprotective Drug Discovery From Phytochemicals and Metabolites for CNS Viral Infection: A Systems Biology Approach With Clinical and Imaging Validation
Source: Front Neurosci. 2022 Jul 25;16:917867. doi: 10.3389/fnins.2022.917867 (PMC9358258; doi:10.3389/fnins.2022.917867)
Supplement: Supplementary file 1 [file Data_Sheet_1.docx]

Neuroprotective Drug Discovery From Phytochemicals and Metabolites for CNS Viral Infection: A Systems Biology Approach With Clinical and Imaging Validation

Supplementary Material

# S1. Image acquisition

A diffusion tensor image (DTI) was acquired using an echo-planar imaging sequence with b values 1000 s/mm^2^ and one reference volume with b =0 s/mm^2^. The DTI acquisition parameters were repetition time 9386 ms, echo time 58 ms, a field of view 224x224, slice thickness of 1 mm, 160 number of slices, and 90° flip angle. A turbo field echo sequence was used to acquire the T1 weighted MRI volume. The scanner acquisition parameters were echo time 4 ms, repetition time 8.5 ms, voxel size of 1x1x1mm^3^, a field of view 240x240x160, slice thickness of 2 mm, 70 number of slices, and 8° flip angle.

# Supplementary Tables and Figures

## 2.1 Supplementary Tables

**Table S1.** The comparison of binding energy and inhibitory constant (Ki) value of ACE inhibitors against inhibitor bound human angiotensin converting enzyme-related carboxypeptidase (ACE2) (PDB ID: 1R4L) and SARS-CoV-2 spike receptor-binding domain bound to the ACE2 receptor (PDB ID: 6M0J).

| Compounds | Site A ( PDB ID: 1R4L) | | | Site B (PDB ID: 6M0J) | | |
| --- | --- | --- | --- | --- | --- | --- |
|  | **Total binding energy**  **(kcal/mol)** | **Calculated inhibitory constant (Ki-Molar)** | **Interacting amino acid residues** | **Total binding energy**  **(kcal/mol)** | **Calculated inhibitory constant (Ki- Molar)** | **Interacting amino acid residues** |
| MLN-4760 | -8.11 | 1.13×10^-6^ | Arg273 His345  Pro346 Thr371 | -2.62 | 1.20×10^-2^ | Gln24 |
| Lisinopril | -8.15 | 1.07×10^-6^ | Arg273 His345  Pro346 Thr371 | -2.16 | 2.59×10^-2^ | Gln24 |

**Table S2.** The increasing order of binding energy and inhibitory constant (Ki) value of phytochemicals, tetracycline class of antibiotics and ceftriaxone against with SARS-CoV-2 spike receptor-binding domain bound to the ACE2 receptor (PDB ID: 6M0J).

| **Compund** | **Total binding energy**  **(kcal/mol)** | **VDW + H bond + desolv Energy**  **(kcal/mol)** | **Calculated inhibitory constant (Ki- Molar)** | **Interacting amino acid residues** |
| --- | --- | --- | --- | --- |
| Chlorogenic acid  Ervacycline | -9.02  -8.23 | -10.48  -9.91 | 2.45×10^-7^  9.26×10^-7^ | Trp436 Ser373 Asn437 Phe342  Gln24 Gln76  Phe28 Glu76 |
| Rolitetracycline | -7.30 | -9.13 | 4.43×10^-6^ | Gln76 Thr27  Lys31 Leu79 |
| Tigecycline | -7.28 | -8.97 | 4.61×10^-6^ | His34 Asn33  Glu37 Phe390 |
| Minocycline | -7.27 | -8.46 | 4.67×10^-6^ | Asp30 His34 Pro389 Phe390 |
| Naringenin  Tetracycline | -7.07  -6.94 | -8.18  -8.32 | 6.57×10^-6^  8.13×10^-6^ | Phe338 Cys336  Leu335 Leu368  Gln24 Tyr63 Met82 Leu79 |
| Quercetin  Doxycycline  Podophyllotoxin  Ceftriaxone | -6.36  -6.19  -5.70  -4.05 | -8.09  -6.87  -8.53  -7.11 | 21.95×10^-6^  29.19×10^-6^  6.62×10^-6^  1.08×10-3 | Cys336 Asp364  Asp30 Leu29 Pro389 Gln96  Gly482 Cys480  Asn481 Pro479  Gln24 Thr27  Lys31 Leu79 |

**Table S3.** The increasing order ofbinding energy and inhibitory constant (Ki) value of phytochemicals, tetracycline class of antibiotics and cephalosporin against COVID-19 main protease (6LU7).

| **Compound** | **Total binding energy**  **(kcal/mol)** | **VDW + H bond + desolv Energy (kcal/mol)** | **Calculated inhibitory constant (Ki- Molar)** | **Interacting amino acid residues** |
| --- | --- | --- | --- | --- |
| Chlorogenic acid  Tigecycline  Podophyllotoxin | -10.01  -8.85  -8.50 | -11.50  -9.89  -11.92 | 45.96×10^-7^  3.25×10^-7^  5.91×10^-7^ | Gln189 Arg188  Met49 Phe140  His41 Gly143  His163 Gln189  Glu166 Gln189  Met49 Asn142 |
| Ervacycline  Quercetin | -8.09  -8.03 | -10.25  -9.73 | 1.18×10^-6^  1.30×10^-6^ | His41 Gly143 Glu166 Pro168  Glu166 Gln189 |
| Minocycline | -7.82 | -10.30 | 1.87×10^-6^ | HIS163 GLN189 |
| Naringenin  Ceftriaxone | -7.68  -6.83 | -8.71  -10.53 | 2.37×10^-6^  9.09×10^-6^ | Gln192 Gln189  His41 Thr190  Gly143 Gln189  His164 His164 |

**Table S4.** The increasing order of binding energy and inhibitory constant (Ki) value of phytochemicals, tetracycline class of antibiotics and cephalosporin against N.S. helicase/ nucleoside triphosphatase of Japanese encephalitis (PDB ID: 2Z83).

| **Compound** | **Total binding energy**  **(kcal/mol)** | **VDW + H bond + desolv Energy (kcal/mol)** | **Calculated inhibitory constant (Ki- Molar)** | **Interacting amino acid residues** |
| --- | --- | --- | --- | --- |
| Ervacycline | -9.81 | -11.36 | 6.48×10^-7^ | Ala264 His266 |
| Tigecycline | -8.48 | -10.84 | 6.11×10^-7^ | Asp542 Pro544 |
| Chlorogenic acid | -7.80 | -9.07 | 1.91 ×10^-6^ | Asp542 Arg600 |
| Minocycline | -7.66 | -9.06 | 2.42×10^-6^ | Asp542 Arg600 |

- 1. **Supplementary Figures on Gene–Gene interaction analysis**


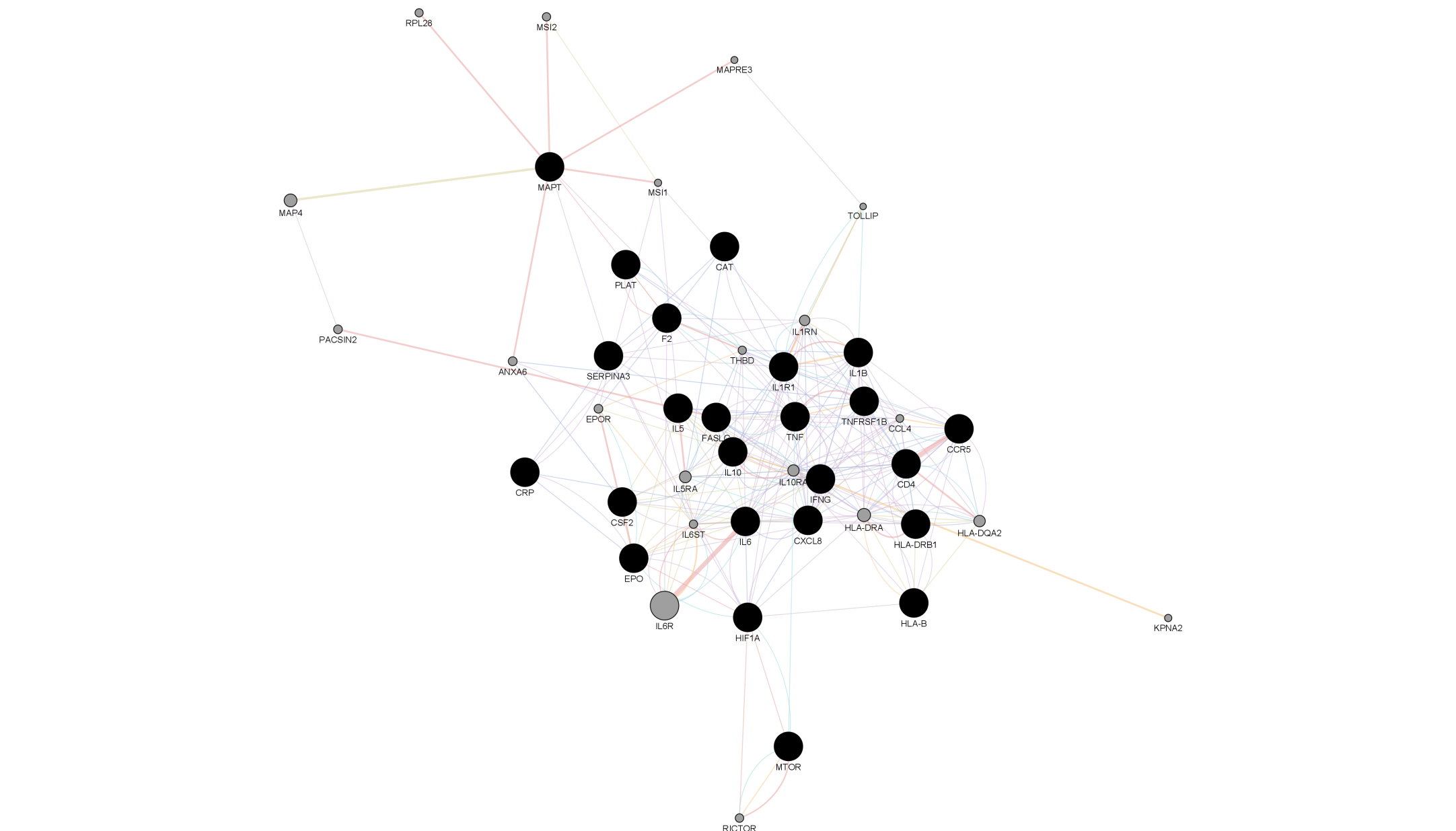


**Supplementary Figure 1a:** GeneMANIA networks showing the gene-gene interaction results of Ceftriaxone targets. Network displays the strength of interaction (edge thickness), type of interaction (colors), many edges in between nodes, and score (size of node).

**Supplementary**
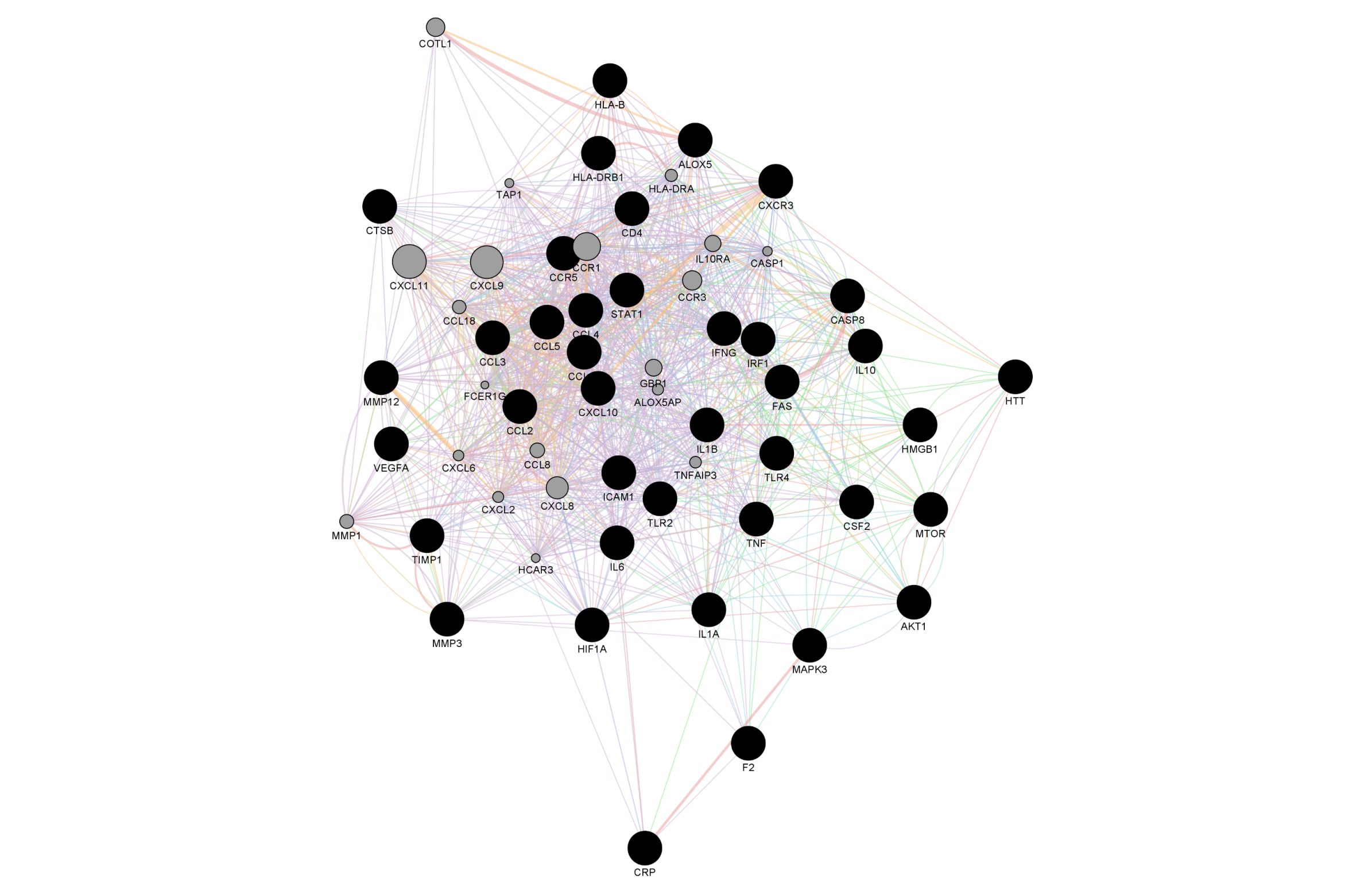
**Figure 1b:** GeneMANIA networks showing the gene-gene interaction results of Minocycline targets. Network displays the strength of interaction (edge thickness), type of interaction (colors), many edges in between nodes, and score (size of node).

**Supplementary Figure 1c:** GeneMANIA networks showing the gene-gene interaction results of Chlorogenic acid targets. Network displays the strength of interaction (edge thickness), type of interaction (colors), many edges in between nodes, and score (size of node).


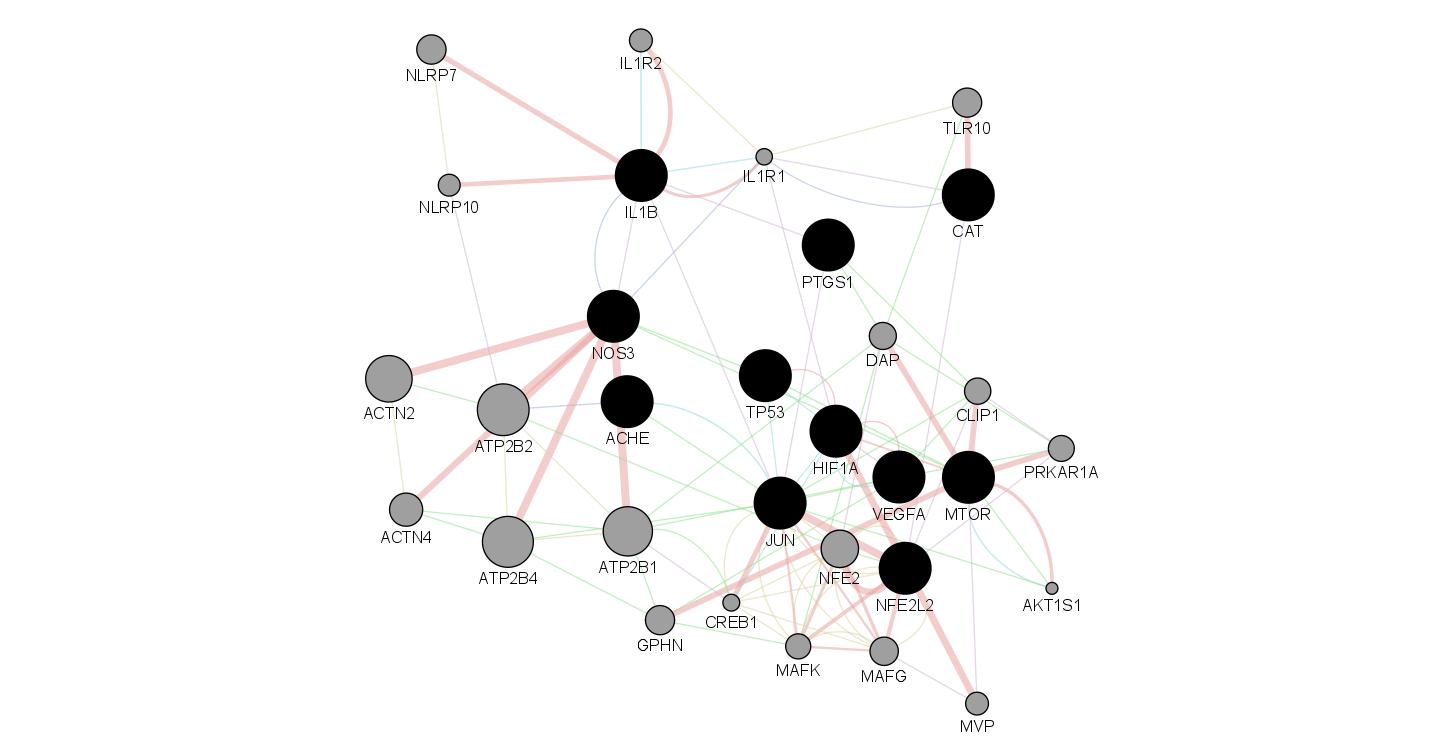


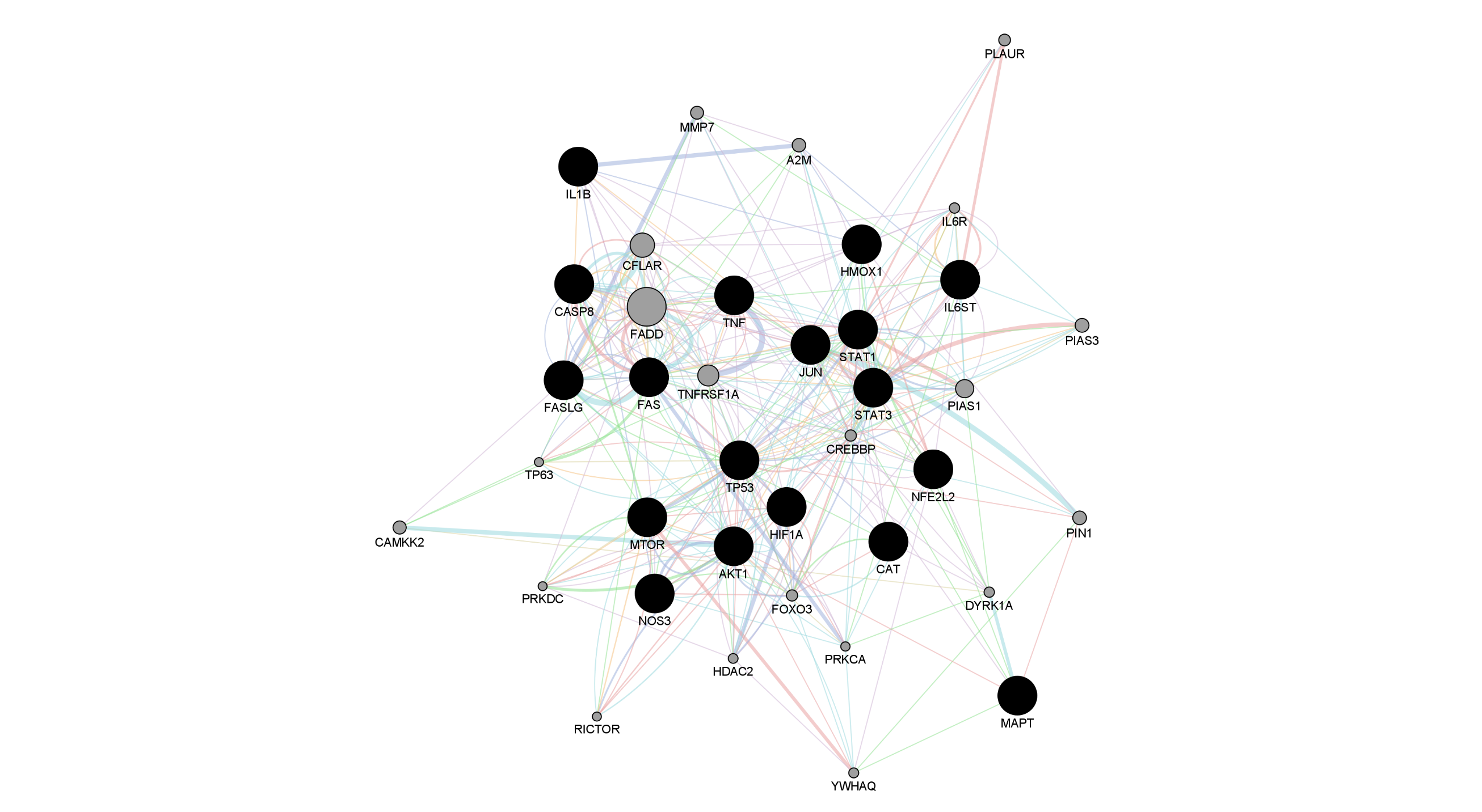


**Supplementary Figure 1d:** GeneMANIA networks showing the gene-gene interaction results of Naringenin targets. Network displays the strength of interaction (edge thickness), type of interaction (colors), many edges in between nodes, and score (size of node).

**Supplementary Figure 1e:** GeneMANIA networks showing the gene-gene interaction results of Podophyllotoxin targets. Network displays the strength of interaction (edge thickness), type of interaction (colors), many edges in between nodes, and score (size of node).

**
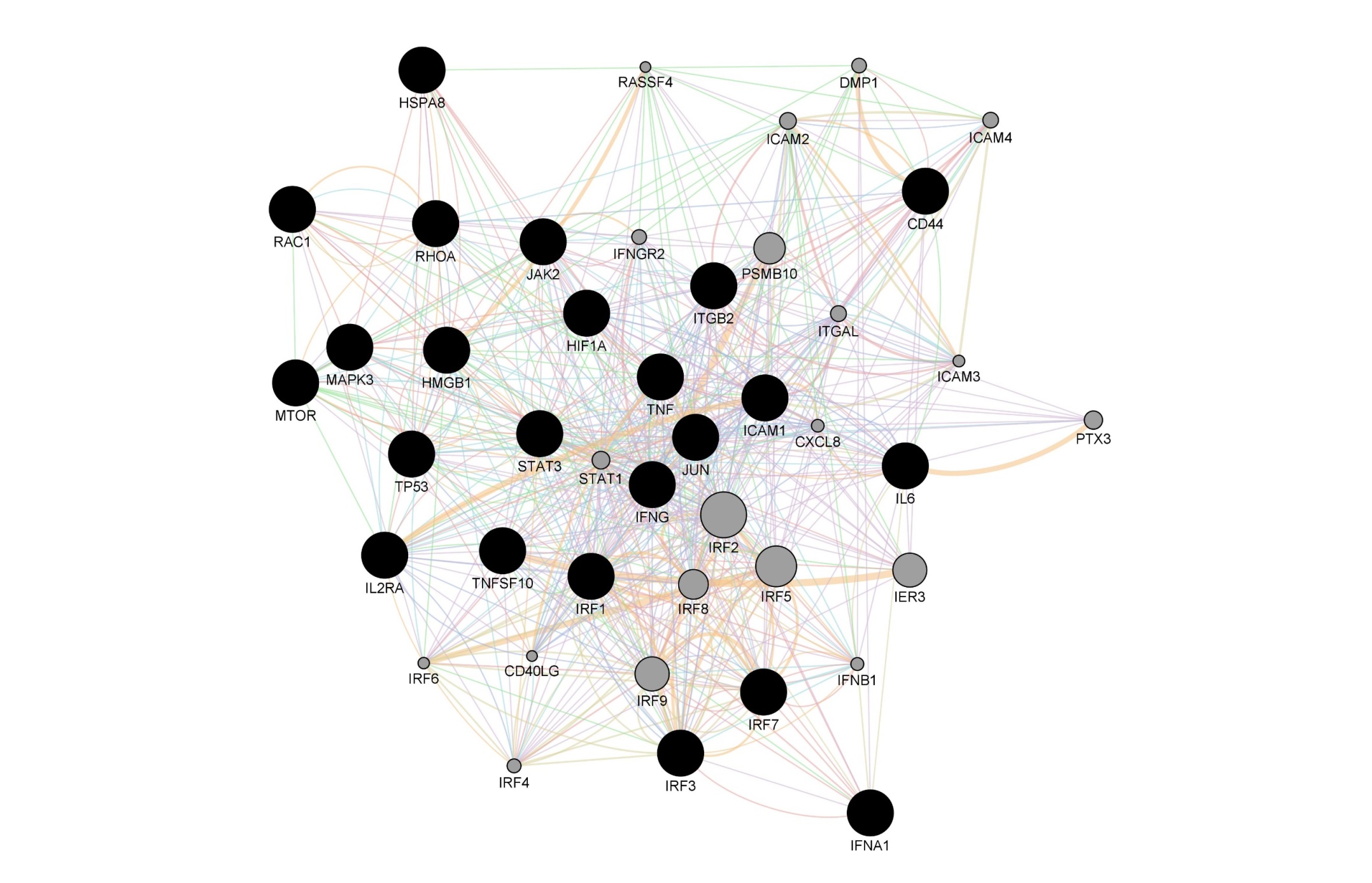
**

- 1. **Supplementary Figures of chemical structures:**











**Chlorogenic acid**

**Naringenin**

**Quercetin**

**Podophyllotoxin**












**Doxycycline**

**Tetracycline**

**Ervacycline**

**Minocycline**










**Tigecycline**

**Ceftriaxone**

**Rolitetracycline**

- 1. **Supplementary figures on re-docked poses**

**
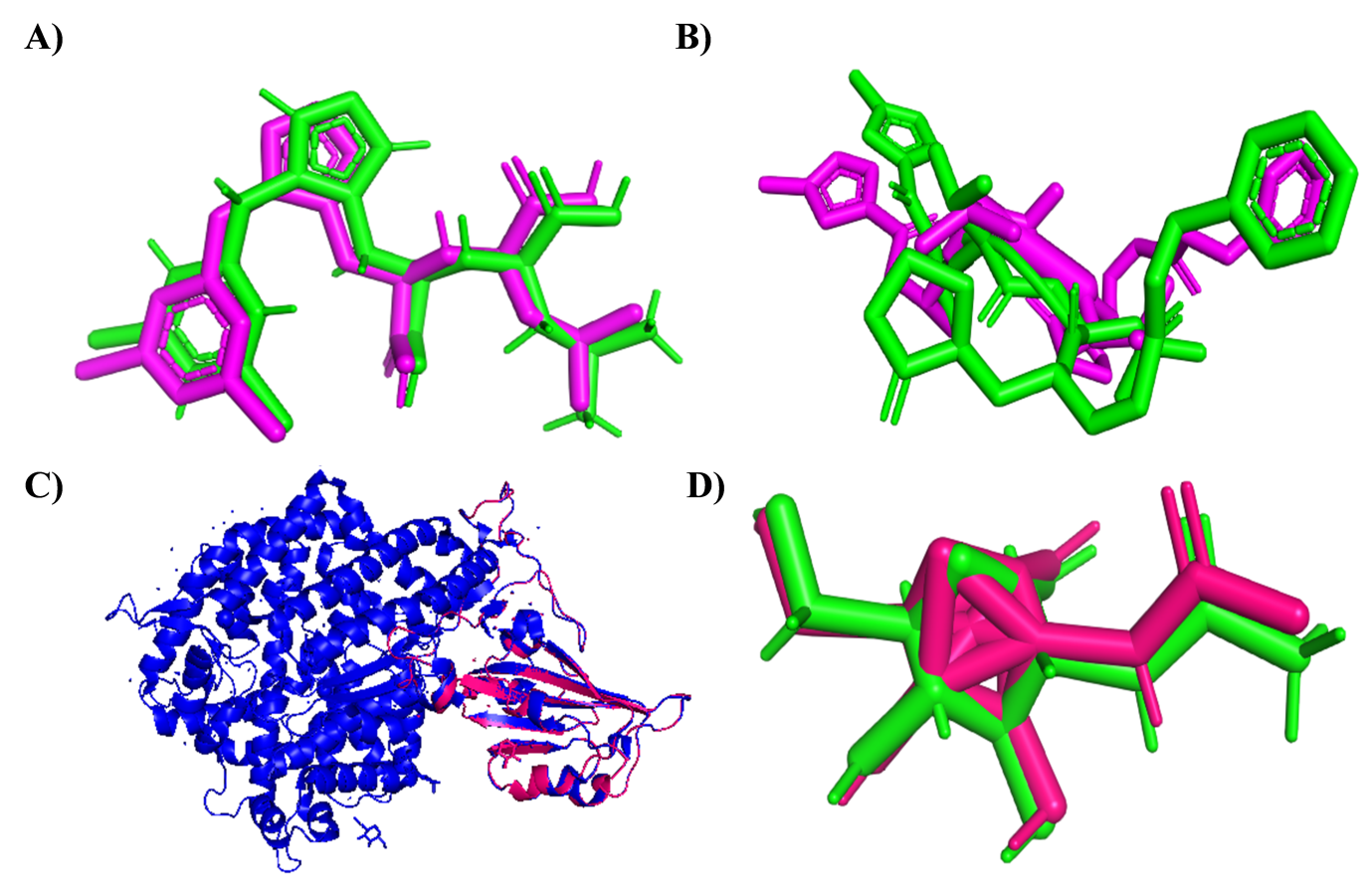
**

**Supplementary Figure 1.4: (A)** Validation experiment showing re-docked and co-crystallized poses of (S,S)-2-{1-carboxy-2-[3-(3,5-dichloro-benzyl)-3h-imidazol-4-yl]-ethylamino}-4-methyl-pentanoic acid on 1R4L.

**(B)** Validation experiment showing re-docked and co-crystallized poses of (N-[(5-methylisoxazol-3-yl)carbonyl]alanyl-l-valyl-n~1~-((1r,2z)-4-(benzyloxy)-4-oxo-1-{[(3r)-2-oxopyrrolidin-3-yl]methyl}but-2-enyl)-l-leucinamide on 6LU7.

**(C)** Validation experiment showing re-docked and bound protein on 6M0J.

**(D)** Validation experiment showing re-docked and co-crystallized poses of 2-acetamido-2-deoxy-beta-D-glucopyranose on 6M0J.
